# Discovery and characterization of small molecules targeting the DNA-binding ETS domain of ERG in prostate cancer

**DOI:** 10.18632/oncotarget.17124

**Published:** 2017-04-15

**Authors:** Miriam S. Butler, Mani Roshan-Moniri, Michael Hsing, Desmond Lau, Ari Kim, Paul Yen, Marta Mroczek, Mannan Nouri, Scott Lien, Peter Axerio-Cilies, Kush Dalal, Clement Yau, Fariba Ghaidi, Yubin Guo, Takeshi Yamazaki, Sam Lawn, Martin E. Gleave, Cheryl Y. Gregory-Evans, Lawrence P. McIntosh, Michael E. Cox, Paul S. Rennie, Artem Cherkasov

**Affiliations:** ^1^ Vancouver Prostate Centre and the Department of Urologic Sciences, University of British Columbia, Vancouver, BC V6H 3Z6, Canada; ^2^ Department of Biochemistry and Molecular Biology, Department of Chemistry, Michael Smith Laboratories, University of British Columbia, Vancouver, BC V6T 1Z3, Canada; ^3^ Department of Ophthalmology and Visual Sciences, Eye Care Centre, University of British Columbia, Vancouver, BC V5Z 3N9, Canada

**Keywords:** prostate cancer, ERG, rational drug design, small molecule inhibitor, TMPRSS2-ERG

## Abstract

Genomic alterations involving translocations of the ETS-related gene *ERG* occur in approximately half of prostate cancer cases. These alterations result in aberrant, androgen-regulated production of ERG protein variants that directly contribute to disease development and progression. This study describes the discovery and characterization of a new class of small molecule ERG antagonists identified through rational *in silico* methods. These antagonists are designed to sterically block DNA binding by the ETS domain of ERG and thereby disrupt transcriptional activity. We confirmed the direct binding of a lead compound, VPC-18005, with the ERG-ETS domain using biophysical approaches. We then demonstrated VPC-18005 reduced migration and invasion rates of ERG expressing prostate cancer cells, and reduced metastasis in a zebrafish xenograft model. These results demonstrate proof-of-principal that small molecule targeting of the ERG-ETS domain can suppress transcriptional activity and reverse transformed characteristics of prostate cancers aberrantly expressing ERG. Clinical advancement of the developed small molecule inhibitors may provide new therapeutic agents for use as alternatives to, or in combination with, current therapies for men with ERG-expressing metastatic castration-resistant prostate cancer.

## INTRODUCTION

Although confounded by disease heterogeneity, the emergence of genome-wide analytics has begun to reveal the spectrum of recurrent genomic alterations that may directly affect prostate cancer (PCa) disease progression and outcome [1, 2]. The first identified, and most prevalent genetic irregularities, occurring in ~50% of PCa patients, involves inter- or intrachromosomal rearrangements that result in fusion of the androgen receptor (AR) DNA response element of transmembrane protease serine 2 (*TMPRSS2*) to variable open reading frames of the ETS family member, ETS-related gene *ERG* [3]. Normally *ERG* is not expressed by prostatic epithelial cells, but fusion with *TMPRSS2* promoter causes aberrant AR-driven *ERG* expression making it one of the most commonly overexpressed genes in PCa. ERG is known to normally regulate endothelial and hematopoietic cell differentiation [4], and has been implicated as a driver of subsets of Ewing's sarcomas, leukemias and primitive neuroectoderm tumors [5]. Detection of *TMPRSS2-ERG* rearrangements in approximately 20% of high-grade prostatic intraepithelial neoplasias suggest that these translocations occur early in disease [6]. Whereas the clinical implication of *TMPRSS2-ERG* rearrangements in PCa remain controversial, many studies have linked patients with these rearrangements with poor disease outcome [7, 8]. Such observations have spurred efforts to establish non-invasive screens for *TMPRSS2-ETS* translocations alone or in conjunction with other prostatic biomarkers to improve risk stratification of men diagnosed with PCa [9, 10].

ERG has been implicated as an oncogenic hub that modulates PCa-associated phenotypes, including disruption of the epithelial differentiation program via AR dysregulation [11], activation of c-Myc, epigenetic reprogramming via EZH2 [12] and promotion of genomic instability via PARP dysregulation [13]. Furthermore, ERG overexpression results in transcriptional reprogramming of prostate epithelium that promotes epithelial-mesenchymal transition (EMT) and enables the transformed cells to acquire migratory and invasive characteristics [11, 14]. Additionally, ERG expression is frequently reactivated in castration-resistant PCa (CRPC) and has been associated with an increased chance of resistance to taxane therapies [15, 16]. Whereas ERG expression is initially driven by the AR via fusion with androgen-responsive promoters, self-driven, feed-forward regulation of the remaining wild-type *ERG* allele has been reported [17]. Recently, inactivating mutations in the E3 ubiquitin ligase *SPOP* has also been indicated to decrease ERG degradation, leading to elevated levels of wild-type ERG [18, 19]. Thus, accumulation of the mutant and wild-type ERG proteins, initially driven by AR, that later become self-sustained when in conjunction with mutations in *SPOP*, presents a new mechanism that may contribute to resistance against AR pathway inhibitors and, through sustained induction of EMT, to disease progression in CRPC.

Evidence that ERG expression in PCa is a critical factor driving PCa development, progression, and metastasis [7, 8] makes it a promising drug target. The feasibility of direct targeting ERG has been first demonstrated by a small molecule, YK-4-279, identified from surface plasmon resonance screening of a small-molecule collection from the National Cancer Institute Drug Targeting Program to disrupt the binding of RNA Helicase A to ETS factor, FLI1, in Ewing's sarcoma [20]. YK-4-279 has also been reported to antagonize ERG activity, although its exact ETS binding mode has yet to be determined [21]. While YK-4-279 is in clinical development for Ewing's sarcoma, issues related to toxicity and pharmacokinetics have been reported [22]. Thus, there are currently no approved drugs that directly target ERG or any other member of the ETS family [4, 23].

We hypothesized that the use of rational drug design approach, supported by *in vitro* and *in vivo* screening methods, could identify small molecules that directly target the DNA-binding ETS domain of ERG and thereby inhibit metastatic potential of ERG-positive PCa. Here we report use of an established drug discovery pipeline [24] that combines *in silico* prediction with *in vitro* and *in vivo* experimentation to identify a new class of anti-ERG compounds. We demonstrate that a lead anti-ERG compound, VPC-18005, inhibits ERG-induced transcription and interacts directly with the ERG-ETS domain, and disrupts the ERG binding to DNA. In addition, the compound reduces migration and invasion rates of ERG-overexpressing cells, and inhibits metastasis in zebrafish xenograft models. These results demonstrate that the discovered compound and its derivatives can be developed as therapeutic options for mitigating disease progression in men with ERG-expressing prostate cancer and ultimately lead to improved survival for men with advanced disease.

## RESULTS

### Discovery of small molecules that target the DNA-binding ETS domain of ERG protein

There are numerous *TMPRSS2-ERG* fusions that encode for *ERG* transcripts. Whereas, the majority produce amino terminal-truncated ERG proteins, all retain the C-terminal DNA-binding ETS domain [8]. This DNA-binding ETS domain is essential for ERG to function as a direct transcriptional regulator, and structural data is available for its complex with DNA. Thus, we reasoned that we could use *in silico* approaches to identify small molecules targeting the ETS domain. Such molecules should therefore inhibit transcriptional activity of all functional ERG mutant proteins by antagonizing their ability to interact with DNA. This in turn might disrupt ERG-mediated transformational events involved in PCa disease development and progression.

**Figure 1:**
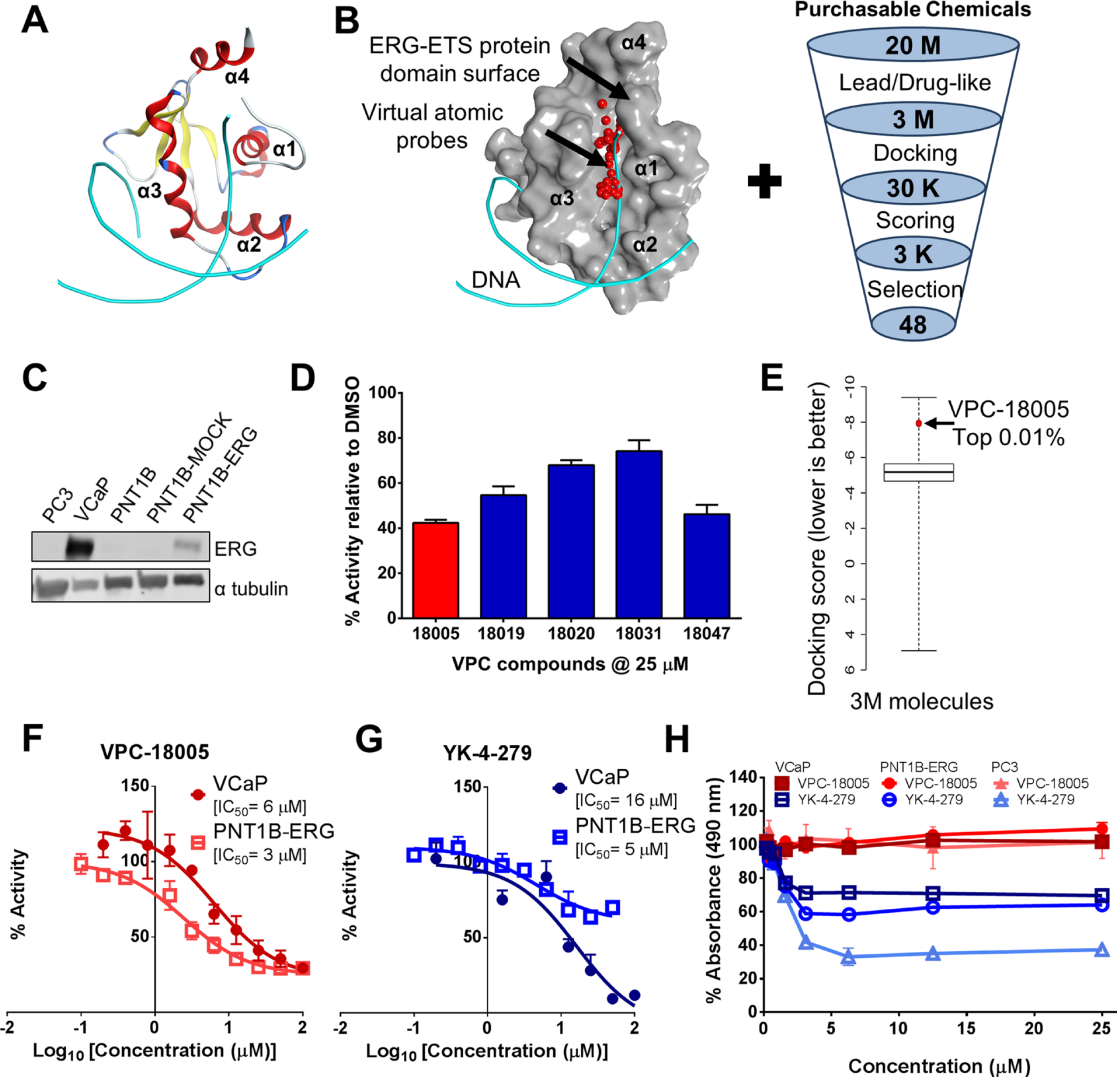
ERG as a drug target and discovery of VPC-18005 (**A**) A ribbon representation of the ERG-ETS domain/DNA complex crystal structure [PDB ID: 4IRI] highlighting the winged helix-turn-helix motif of the ETS domain with helix α3 (red) positioned within the major groove of the DNA (cyan). (**B**) Left: The ERG-ETS domain pocket (shown as grey molecular surface) that was identified by virtual atomic probes (red spheres) and used to screen 3 million small molecules from the ZINC database. The DNA backbone (cyan) is shown for illustration purposes, but not included in virtual screening. Right: Virtual screening pipeline highlighting the steps taken to identify the top candidates to move forward into *in vitro* experiments (M = million; K = thousands). (**C**) Western blot analysis of lysates from the indicative prostate cancer cell lines. Levels of ERG (upper panel) are shown relative to alpha-tubulin as a loading control (lower panel). (**D**) Luciferase activity of lead candidate VPC-18005 (red bar) at 25 μM is shown against other compounds identified from the virtual screening. Data are presented as the mean ± SEM of 4 technical replicates and expressed as a percentage of luciferase activity relative to DMSO control. (**E**) A box plot illustrates the distribution of docking scores for 3 million small molecules docked at the ERG-ETS pocket, and VPC-18005 scored in the top 0.01%. (**F**) Dose response effect of VPC-18005 (media concentration 0.1–100 μM) in PNT1B-ERG (open square) and VCaP (closed circle) cells on ERG-mediated luciferase activity, with IC_50_ (half-maximal inhibitory concentrations) values of 3 μM and 6 μM, respectively. Data are presented as the mean ± SEM of 4 technical replicates and expressed as a percentage of luciferase activity (Luciferase/Renilla) relative to DMSO control. Data were fitted using GraphPad Prism 6 software to calculate dose response curves of log_10_ (inhibitor concentration) vs response. (**G**) Dose response effect of YK-4-279 (media concentration 0.1 – 100 μM) in PNT1B-ERG (open square) and VCaP (closed circle) cells on ERG-mediated luciferase activity, with IC_50_ values of 5 μM and 16 μM, respectively. Data from 4 technical replicates are presented and fit as explained in Figure 1F. (**H**) Cell viability (MTS) of ERG-expressing cells (PNT1B-ERG (circle) and VCaP (square)) and non-ERG expressing cells (PC3 (triangle)) after treatment with 0.2 to 25 μM VPC-18005 (closed red shape) or published inhibitor YK-4-279 (open blue shape) for 72 h. Impact on viability is presented as the mean ± SEM of 3 technical replicates and expressed as a percentage of absorbance relative to DMSO control.

A structure-based virtual screening approach, previously established for targeting protein-DNA and protein-protein interaction interfaces [24], was applied to the 1.7Å resolution ERG-ETS domain crystal structure [PDB ID: 4IRG] [25]. The DNA binding interface was identified from a 2.8 Å resolution crystal structure of the corresponding ERG-DNA complex [PDB ID: 4IRI] (Figure 1A). The ERG-ETS domain contains a winged helix-turn-helix motif, with helix α3 positioned within the major groove of the DNA containing a cognate GGAA sequence [25]. A top-ranked druggable surface pocket was identified by virtual atomic probes to partially overlap this ERG-DNA interface (Figure 1B). The identified pocket is adjacent to the DNA recognition helix (α3), and thus it was predicted that a small molecule bound at this site will competitively block DNA binding. Three million chemical structures derived from the ZINC database [26] were individually docked into this pocket. Combining the docking scores, binding poses, consensus voting and drug-like properties (detailed in Materials and Methods), an initial set of 48 compounds, representing 45 different chemical classes, were selected for *in vitro* analysis.

To evaluate the biological anti-ERG activity of the compounds identified above, we first assessed ERG expression in a panel of prostate cell lines (Figure 1C). We confirmed expression of the ERG protein in VCaP (endogenous overexpression) and PNT1B-ERG cells (stable ERG overexpression [14]). In contrast, PC3, PNT1B and PNT1B-Mock cells were negative for ERG expression [14, 27]. Each of the compounds was first evaluated in PNT1B-ERG cells at concentrations of 10 μM and 25 μM for its ability to inhibit ERG transcriptional activation of a transiently transfected, endoglin E3 promoter-derived [28], ETS-responsive firefly luciferase reporter (pETS-luc) construct containing 3 conserved ETS recognition (GGAA) motifs. A representative example of 5 compounds that showed suppression of the luciferase reporter by 20%-60% are identified in Figure 1D. Compound VPC-18005 was identified as the most potent inhibitor of luciferase activity from this initial set. The molecular docking score of VPC-18005 was ranked in the top 0.01% of all 3 million molecules evaluated in the virtual screening discussed earlier (Figure 1E). Before proceeding with in-depth analysis, the media solubility and stability of VPC-18005 were assessed (Supplementary Figure 1). VPC-18005 was soluble in media and remained stable for at least 3 days (93%). For comparison, the published inhibitor YK-4-279 was soluble but less stable (60%). A more thorough dose response analysis was performed using both VCaP and PNT1B-ERG cells to evaluate the potency of VPC-18005. VPC-18005 was found to inhibit pETS-luc reporter activity in PNT1B-ERG and VCaP cells with IC_50_ values of 3 and 6 μM, respectively (Figure 1F). For comparison, YK-4-279 [21] exhibited IC_50_ values of 5 μM and 16 μM in PNT1B-ERG and VCaP cell-based ETS-Luc reporter assays, respectively (Figure 1G).

In order to assess whether the suppressed pETS-luc reporter activity was due to cytotoxicity, an MTS assay was performed over 72 hours (h) to measure the impact of the compounds on cell viability (Figure 1H). VPC-18005 treatment (0.2–25 μM) did not decrease viability of either ERG-expressing cells (PNT1B-ERG and VCaP) or non-ERG expressing (PC3) prostatic cells. Previous reports suggest that the IC_50_ for YK-4-279 cytotoxicity is 10 μM in VCaP cells and > 100 μM in PC3 cells [21]. However in this study we observed YK-4-279-mediated inhibition of cell viability in both ERG expressing and non-ERG expressing cell lines at doses ≥ 5 μM (Figure 1H). Cell cycle analysis was conducted and confirmed the results of the viability assay (Supplementary Figure 2A). Whereas VPC-18005 did not impact cell cycle distribution, YK-4-279 substantially increased the sub-G_0_ population at 5 and 10 μM doses (*p* = 0.003 and 0.003, respectively). The G_0_–G_1_ phase population was concomitantly reduced after 5 and 10 μM YK-4-279 treatment (*p* = 0.003 and 0.006, respectively). Real time cell analysis showed that VPC-18005 did not suppress proliferation or induce cell death (Supplementary Figure 2B). A dose-dependent effect of YK-4-279 on cell growth was observed (Supplementary Figure 2C), and the cells were observed to have altered morphology, indicative of toxicity and cell death.

To further assess if VPC-18005 has any non-specific cellular effect, luciferase assays were performed in PNT1B-MOCK and -ERG cells treated with increasing concentrations of VPC-18005 (Supplementary Figure 3A), and VPC-18005 had minimal impact on the reporter signal in PNT1B-MOCK as compared to PNT1B-ERG cells. Furthermore, overexpression of ERG protein through R1881 treatment counteracted VPC-18005 inhibition in the luciferase assay (Supplementary Figure 3B). VPC-18005 was also tested against an androgen receptor luciferase reporter (ARR_3_tk-luc) and showed no significant effect on the reporter expression (Supplementary Figure 3C). Collectively, these results indicated that VPC-18005 could suppress ERG reporter activity without exhibiting overt cytotoxicity.

### Direct binding of VPC-18005 to the ERG-ETS domain

**Figure 2:**
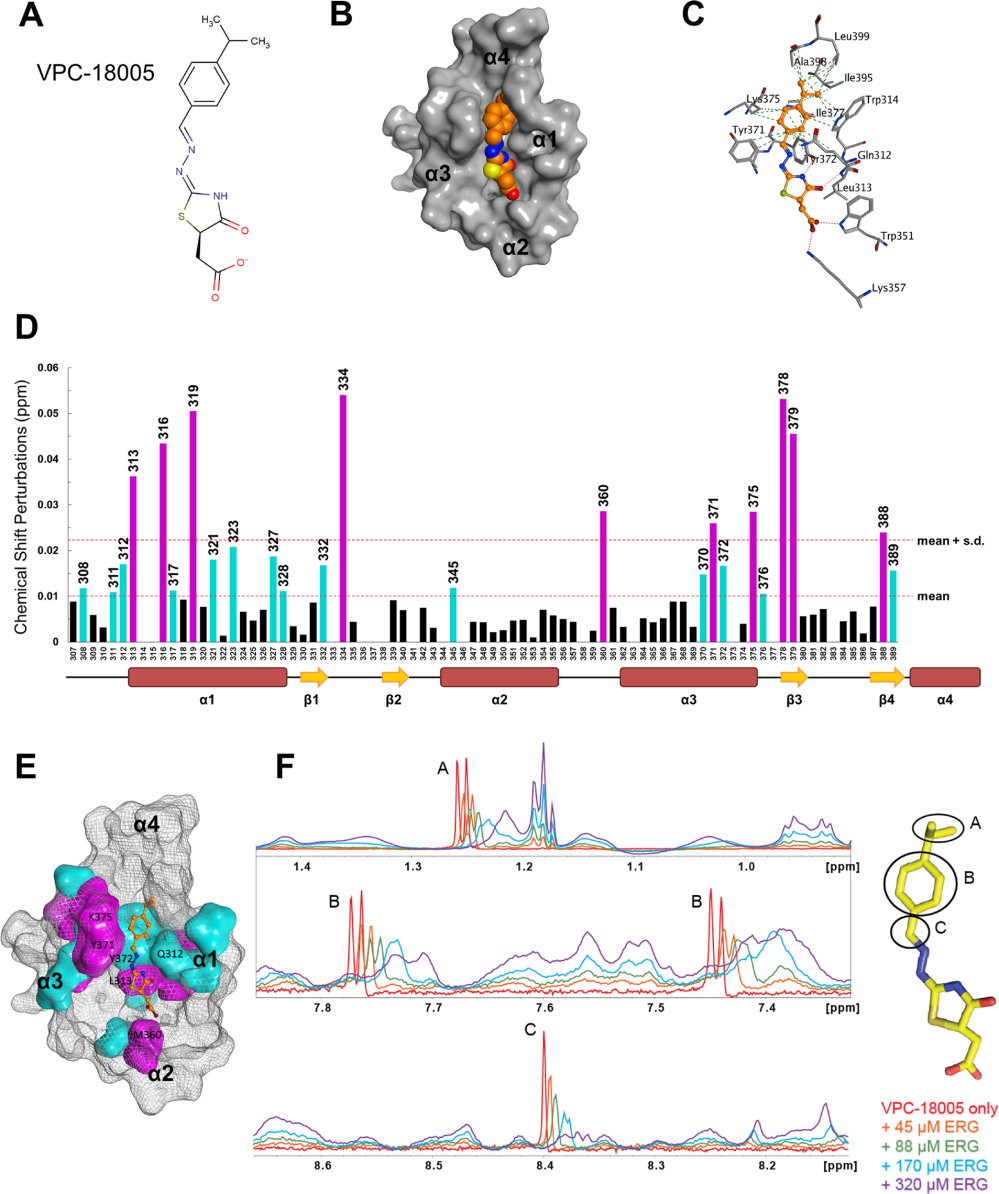
Characterization of VPC-18005 binding to the ERG-ETS domain (**A**) Chemical structure of VPC-18005, in the isomeric form used for docking. The R-isomer is calculated to have the most favorable binding energy. (Molecular weight = 318 g/mol at pH 7). (**B**) A space-filling representation of the predicted VPC-18005 binding pose within the ERG-ETS domain pocket (orange = carbon, blue = nitrogen, red = oxygen, yellow = sulfur). (**C**) Protein residues that are predicted to interact with VPC-18005 at the ERG-ETS domain. The red dotted lines indicate hydrogen bonds, and the green lines represent non-polar packing interactions. (**D**) Amide chemical shift perturbations resulting from the addition of a 10 fold molar excess of VPC-18005 to the ERG-ETS domain (derived from Supplementary Figure 5). Coloured bars denote significant changes (magenta ≥ mean + standard deviation, cyan ≥ mean). The secondary structure of the EGR-ETS domain is shown at the bottom. (**E**) Amino acid residues exhibiting significant chemical shift perturbations were mapped to their corresponding locations on the ERG-ETS domain (same colour code as in D). (**F**) ^1^H-NMR monitored titration of VPC-18005 (sharp signals) with increasing concentrations (red through purple) of the ERG-ETS domain (broad signals). Signals from ^1^H nuclei directly bonded to the indicated chemical moieties shift and broaden upon binding the protein.

The chemical structure of VPC-18005 is depicted in Figure 2A. Using computational modeling methods, the predicted binding pose of VPC-18005 was visualized in more detail inside the target pocket on the ERG-ETS domain (Figure 2B and 2C). VPC-18005 is composed of a hydrophobic isopropyl benzyl group at one end and a negatively charged 5′ carboxyl 4-thiazolidanone group on the other end, linked by an azo moiety with conjugated double bonds. Within the binding pocket on the ERG-ETS domain, VPC-18005 is predicted to form a salt bridge with Lys357, hydrogen bonds with Leu313, Trp351 and Tyr372, and hydrophobic interactions with a number of surrounding amino acid residues, including Gln312, Trp314, Tyr371, Tyr372, Lys375, Ile377, Ile395, Ala398, and Leu399 (residue numbering based on ERG isoform 5, UniProt ID: P11308-4; Figure 2C).

We utilized NMR spectroscopy to directly assess the binding of VPC-18005 with the ERG-ETS domain. The ^15^N-HSQC spectrum of ^15^N-labelled protein (100 μM) was assessed in the presence of increasing concentrations of DMSO-solubilized VPC-18005 (Supplementary Figure 5A and 5B), as well as with a DMSO control (Supplementary Figure 5D and 5E). The spectra demonstrated small dose-dependent chemical shifts changes for a number of amide ^1^H^N^-^15^N groups that occurred upon addition of VPC-18005, but not DMSO. A chemical shift perturbation plot with VPC-18005 at 1:10 molar ratio (i.e. 1 mM) showed that protein residues with changes greater than the mean (0.01 ppm) were mostly located along helix α1, helix α3 and strand β3 (Figure 2D). These amides cluster around the predicted binding pocket of VPC-18005 (Figure 2E), supportive of its binding pose with the ERG protein. Of note, residues with perturbed amide chemical shifts, including Leu313 on helix α1 and Tyr371, Try372, Lys375 on helix α3, modeled to interact with VPC-18005 through hydrogen bonds and hydrophobic interactions, have also been previously shown to be involved in ERG-DNA interactions [25]. Fitting of the ^15^N-HSQC titration curves to a simple 1:1 binding isotherm yielded a K_D_ value of ~3 mM for the interaction of VPC-18005 with recombinant ERG-ETS domain (Supplementary Figure 5C). To further localize the binding interactions between VPC-18005 and the ERG-ETS domain, the reverse titration was performed. In this case, the ^1^H-NMR spectrum of VPC-18005 was monitored vs. increasing concentrations of recombinant ERG-ETS domain. Several ^1^H nuclei of VPC-18005 exhibited ERG-dependent chemical shift perturbations. These include the hydrogens on the aromatic ring (^1^H 7.78 and 7.45 ppm), the methyls on the isopropyl group (^1^H 1.25 ppm) and the conjugated double bond (^1^H 8.4 ppm) (Figure 2F). Due to the spectral overlap with signals from DMSO, perturbations from the CH_2_ group near the carboxyl group of VPC-18005 could not be determined. Overall, these two complimentary direct binding assay results are consistent with the proposed model for how VPC-18005 binds to the ERG-ETS protein domain at the interface required for DNA interaction.

### VPC-18005 disrupts binding of the ERG-ETS domain to DNA

**Figure 3:**
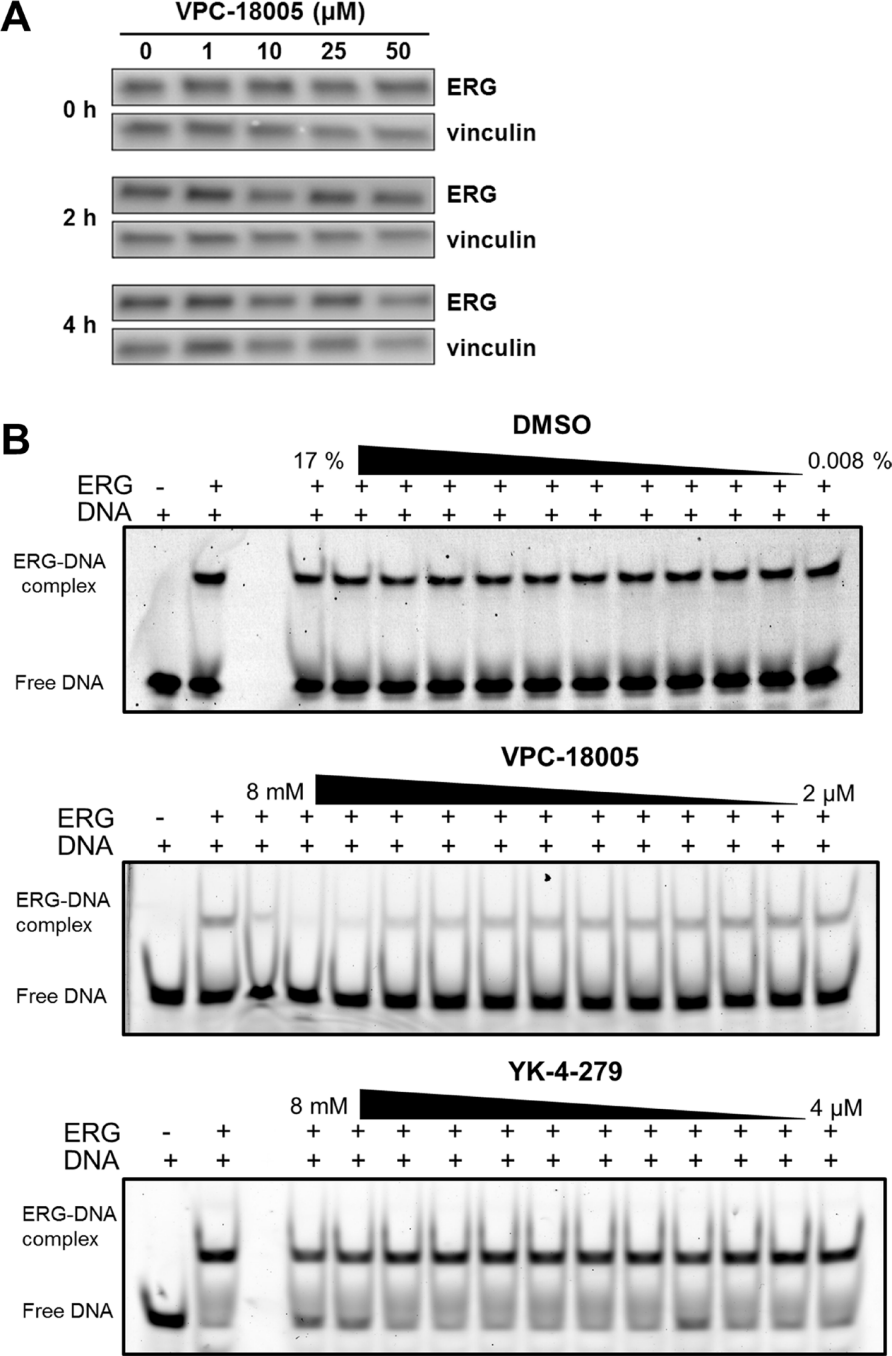
VPC-18005 disrupts binding of the ERG-ETS domain to DNA (**A**) Western blot analysis of ERG expression (upper panel) relative to vinculin (lower panel) in lysates from VCaP cells treated for 1 h with cycloheximide and then cultured for 0, 2 or 4 h with VPC-18005 at the indicated concentrations. (**B**) EMSA shows binding of 4 nM ERG-ETS domain to 1 nM fluorescently-labeled dsDNA alone and in the presence of increasing concentrations of DMSO (top panel, 0.008–17%)), VPC-18005 (middle panel, 2 μM–8 mM), and YK-4-279 (lower panel 4 μM–8 mM).

As there was no obvious effect of VPC-18005 on general cytotoxicity (Figure 1H), we assessed whether the impact of VPC-18005 treatment on ETS reporter activity was due to decreased ERG protein stability. Pre–treatment with the protein synthesis inhibitor cycloheximide did not induce ERG degradation after treatment with VPC-18005 at up to 50 μM for 4 h (Figure 3A). At extended time points of 24 and 48 h, there was still no observable ERG protein degradation (Supplementary Figure 3D).

Since ERG protein levels were stable in cells treated with VPC-18005 and NMR data supported its direct binding to the ERG-ETS domain, we next assessed whether VPC-18005 could disrupt ERG-DNA binding. Electrophoretic mobility shift assays (EMSA) were performed using purified ERG-ETS domain and a DNA oligonucleotide containing the consensus GGAA recognition motif. The recombinant ERG-ETS domain binds this cognate DNA with a K_D_ ~ 1 nM (Supplementary Figure 6A). VPC-18005, but not DMSO control, exhibited dose-dependent disruption of recombinant ERG-ETS/DNA complex formation (Figure 3B) with a K_I_ value of ~250 μM (Supplementary Figure 6B). Although indicative of relatively weak binding, this is in agreement with the K_D_ value determined for the interaction of the ERG-ETS domain and VPC-18005 using ^15^N-HSQC spectroscopy (Supplementary Figure 5C). In contrast, YK-4-279 did not disrupt binding between the ERG-ETS domain and DNA (Figure 3B). These results were further confirmed using VCaP nuclear lysate where VPC-18005, but not YK-4-279, disrupted ERG-DNA complex formation (Supplementary Figure 6C–6E). Collectively, these results indicate that VPC-18005 can disrupt binding of the ERG protein to the DNA containing ETS-response elements.

**Figure 4:**
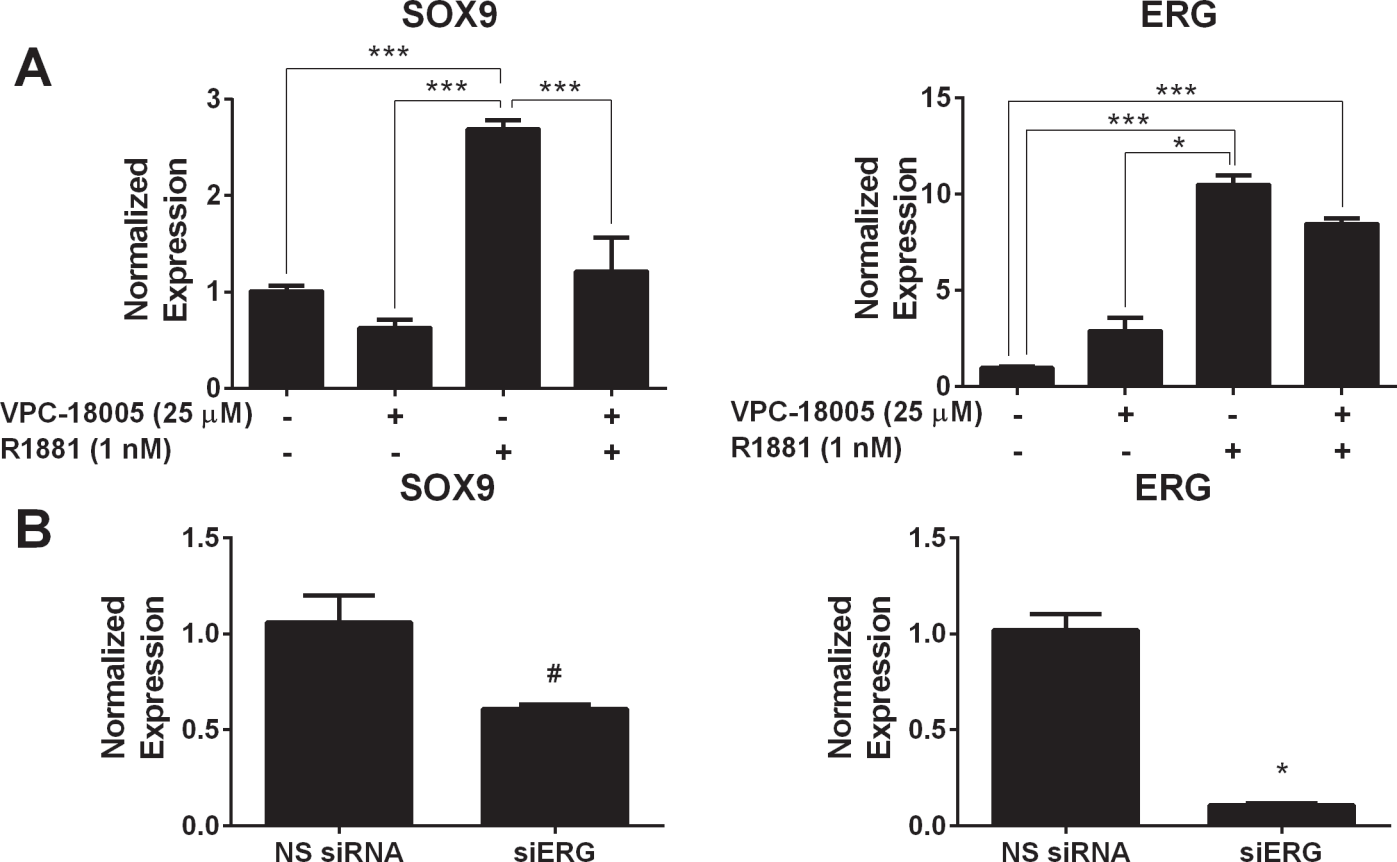
VPC-18005 inhibits SOX9 gene expression (**A**) SOX9 and ERG mRNA levels in VCaP cells treated with and without 1 nM R1881 and 25 μM VPC-18005. ***p < 0.001; *p < 0.05, Kruskal–Wallis test with Dunn's multiple comparison post-hoc test. (**B**) SOX9 and ERG mRNA levels in VCaP cells transfected with ERG siRNA for 48 h. *p < 0.0001, unpaired *t*-test; ^#^ *p* = 0.0007, Mann-Whitney-U test. Expression of ERG and SOX9 was normalized to GAPDH. Data points represent experiment performed in triplicate. Error bars indicate standard error of mean for *n* = 9 values.

A previous study [29] has shown that ERG induces *SOX9* gene expression through an AR-regulated enhancer in VCaP. SOX9, a member of the SOX (SRY-related HMG box) family, is a transcription factor that is required for prostate organogenesis, and its dysregulation has been implicated in cancer pathogenesis [30]. SOX9 overexpression in an LNCaP xenograft mouse model resulted in increased tumor growth and invasion [31], and SOX9 depletion in VCaP was shown to inhibit *in vitro* and *in vivo* invasion [29]. SOX9 is basally expressed in VCaP cells and elevated following Metribolone (R1881) treatment (Figure 4A). Basal and R1881-stimulated SOX9 mRNA and protein expressions were markedly decreased following VPC-18005 treatment. Reduction of *ERG* and *SOX9* expression was also confirmed following siRNA knockdown of *ERG* in VCaP cells compared to non-specific (NS) siRNA control (Figure 4B).

### VPC-18005 inhibits migration and invasion of ERG-overexpressing cells *in vitro*

**Figure 5:**
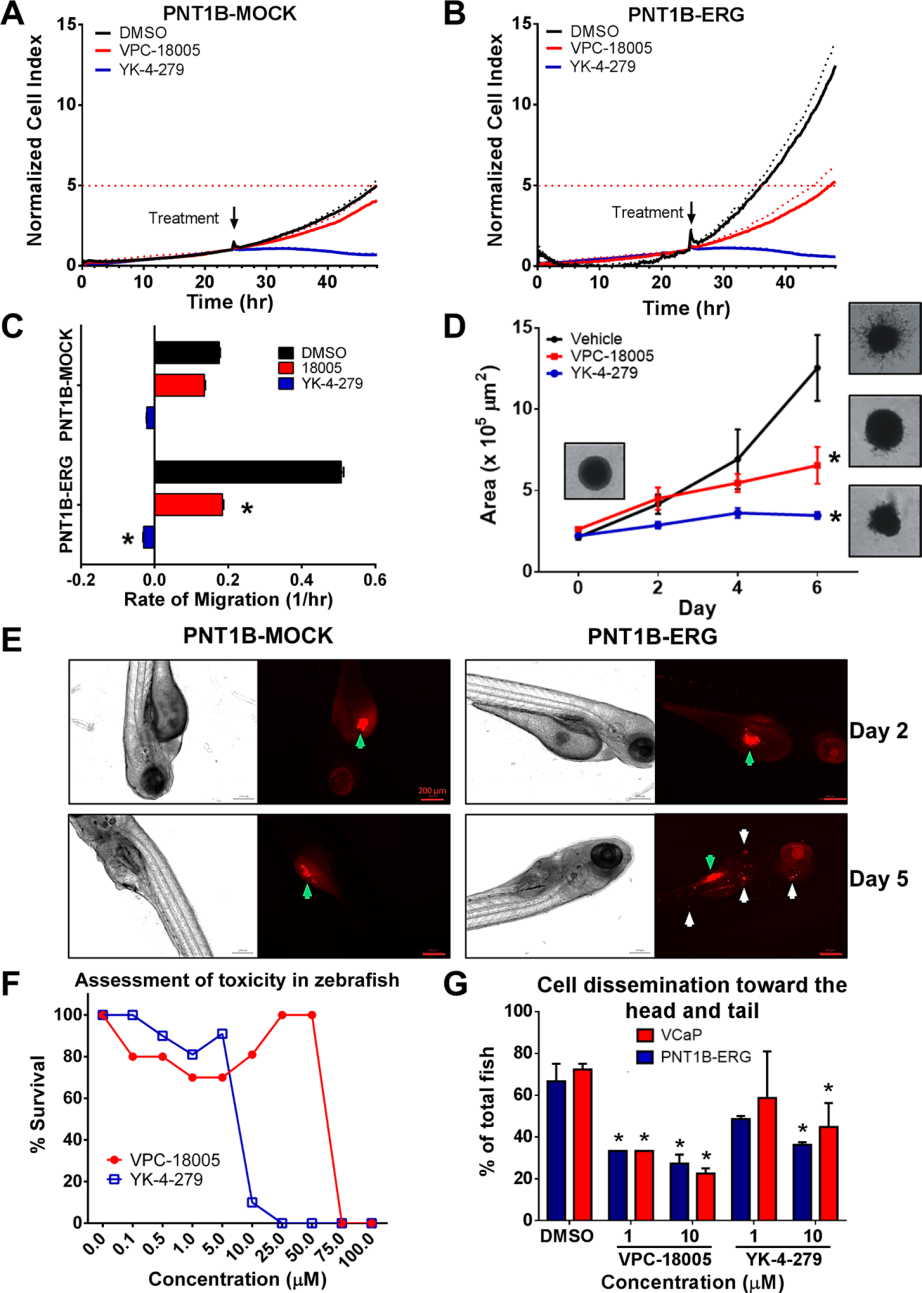
VPC-18005 inhibits migration and invasion of prostate cell lines *in vitro* and *in vivo* (**A**) PNT1B-Mock cells and (**B**) PNT1B-ERG cells were seeded in the upper chamber of a real-time cell analysis system (xCelligence) and treated with 5 μM VPC-18005 (red line), YK-4-279 (blue line) or 0.01% DMSO (control; black line) at 24 h. The normalized cell index is a measure of the migration of the cells through the pores of the upper chamber and is used as the migration index. Dotted lines represent standard deviations (*n* = 3). The horizontal dotted red line indicates the level of migration the PNT1B-MOCK cells reached at 48 h in comparison to -ERG cells. (**C**) Rates of migration were determined by the slopes of the curves between 24–48 h for VPC-18005 (red) (*p* = 0.031, unpaired *t*-test) and YK-4-279 (blue) (*p* < 0.001, unpaired *t*-test) relative to DMSO control (black). (**D**) Quantitative analysis of PNT1B-ERG spheroid invasion into the surrounding matrix in the presence or absence of VPC-18005 (red line), YK-4-279 (blue line), or 0.01% DMSO (black line) over the period of 6 days. The rate of invasion between day 2 and 6 was significantly reduced in those cells treated with VPC-18005 (*p* = 0.02, unpaired *t*-test) and YK-4-279 (*p* = 0.005, unpaired *t*-test) compared to vehicle control. Error bars indicate standard error of the mean (*n* = 3). (**E**) Pre-stained PNT1B-Mock and PNT1B-ERG cells were microinjected into the yolk sac (green arrows) of the zebrafish, and the metastatic capability of the cells (white arrows) were detected using confocal microscope at day 2 and day 5. (**F**) Evaluation of compound toxicity to zebrafish embryos. Zebrafish embryos were treated with increasing concentration of VPC-18005 and YK-4-279 in their water. After 4 days, surviving embryos were counted. (**G**) Following 5 days of daily treatment, VPC-18005 reduced occurrence of metastasis in zebrafish grafted with PNT1B-ERG and VCaP cells. DMSO versus 1 μM (*p* = 0.03/0.03, chi square) and 10 μM (*p* = 0.002/<0.001, chi square) VPC-18005 (PNT1B/VCaP). YK-4-279 was significant only at 10 μM (*p* = 0.02/0.04, chi square).

ERG promotes EMT, which enables cells to acquire migratory and invasive characteristics [7]. We have previously shown that PNT1B cells acquired these invasive characteristics when ERG was stably overexpressed [14]. Therefore, we aimed to determine if VPC-18005 was able to affect migration and invasion of these cells. PNT1B-MOCK and -ERG cells were plated into the upper chamber of a double chamber real-time cell analysis system and treated with VPC-18005 after 24 h. As expected, in the absence of VPC-18005, PNT1B-ERG exhibited an increased rate of migration toward the serum-containing bottom chamber compared to the PNT1B-MOCK control (Figure 5A and 5B). After 24 h exposure and in comparison to a DMSO control, VPC-18005 (5 μM) significantly reduced the rate of migration of the PNT1B-ERG cells relative to vehicle-treated cells, and the resulting migration rate was indistinguishable from that observed for vehicle treated PNT1B-MOCK cells (Figure 5C). In contrast, but consistent with the cytotoxicity results described earlier, treatment with YK-4-279 resulted in cytotoxicity in both cell lines. RWPE prostate cells, engineered to overexpress ERG, were also tested in this assay. No effect on cell viability was observed following treatment with increasing concentrations of VPC-18005 (Supplementary Figure 4A), and VPC-18005 had a moderate effect on RWPE-ERG cell migration compared to MOCK control (Supplementary Figure 4B and 4C). To further explore this inhibitory effect of VPC-18005, PNT1B-ERG spheroids, pretreated for 24 h with vehicle control or VPC-18005, were submerged in matrix in the presence or absence of treatments, and monitored for 6 days (Figure 5D). Analysis of images captured every 2 days revealed that the rate of invasion between day 2 and 6 was significantly reduced in both VPC-18005 (*t*-test; *p* = 0.02) and YK-4-279 (*p* = 0.005) treated cells compared to vehicle control. These results indicated that VPC-18005 inhibited migration and invasion of ERG-overexpressing cells, without inducing cytotoxicity.

### VPC-18005 inhibits metastasis of ERG-overexpressing cells *in vivo*

To determine whether VPC-18005 could affect cell migratory behavior in an animal model, we utilized zebrafish xenotransplantation as a tool to investigate cell extravasation [32]. We first investigated whether PNT1B-MOCK and PNT1B-ERG could disseminate through the zebrafish body (Figure 5E). Fluorescently tagged cells were injected into the yolk sac, and after 5 days PNT1B-ERG could be seen throughout the body of the fish (Supplementary Video 1). In contrast, PNT1B-MOCK cells were not detected outside of the yolk sac. The embryos also remained viable when cultured in the presence of up to 50 μM VPC-18005 for 72 h. In contrast, YK-4-279-treated embryos exhibited toxicity at concentrations > 10 μM (Figure 5F). Yolk sac-inoculated PNT1B-ERG and VCaP cells were found to become disseminated toward the head and tail of 65 to 70% of embryos, respectively. When cultured in the presence of VPC-18005 at 1 and 10 μM, this percentage of fish with PNT1B-ERG or VCaP dissemination was reduced to 20–30% of inoculated animals (Figure 5G). Culturing embryos in YK-4-279 at 1 and 10 μM resulted in yolk sac dissemination in 40–60% of inoculated animals (Figure 5G). These assays provide first principle evidence that small molecules such as VPC-18005 can antagonize the metastatic potential of ERG-expressing prostate cells.

## DISCUSSION

The ETS family of transcription factors are important targets for drug development because of their strong implications in numerous cancers [4]. However, targeting these transcription factors with small molecules is a challenging task due to their lack of “druggable” active sites. ERG is an important therapeutic target in PCa. We confirmed *ERG* overexpression in PCa by comparing tumor-specific upregulated genes from three published datasets [1, 33, 34] based on a 2-fold differential expression threshold (Supplementary Figure 7). Whereas there were a number of genes dysregulated in each pair-wise dataset comparison (Supplementary Tables 1 and 2), the only upregulated gene common in all three datasets was *ERG*. This highlights ERG as a potential major influencer of PCa. There are currently several reports describing efforts to target ERG with various agents, but none have resulted in approved therapeutics. These include the use of siRNA [35, 36], shRNA [37], peptidomimetics [38] and a small molecule, DB1255 that interacts not with the ERG protein but rather the ETS recognition site on the DNA (GGAA) [39]. In addition, ERG has been targeted indirectly through inhibition of ERG binding proteins including PARP1 [13] and USP9X [40], as well as via ERG-regulated genes, such as YAP1 [41]. As noted earlier, among the various efforts to antagonize ERG, only one small molecule, YK-4-279, has been reported to directly target the ERG protein [22], but with limitations detailed above.

**Figure 6:**
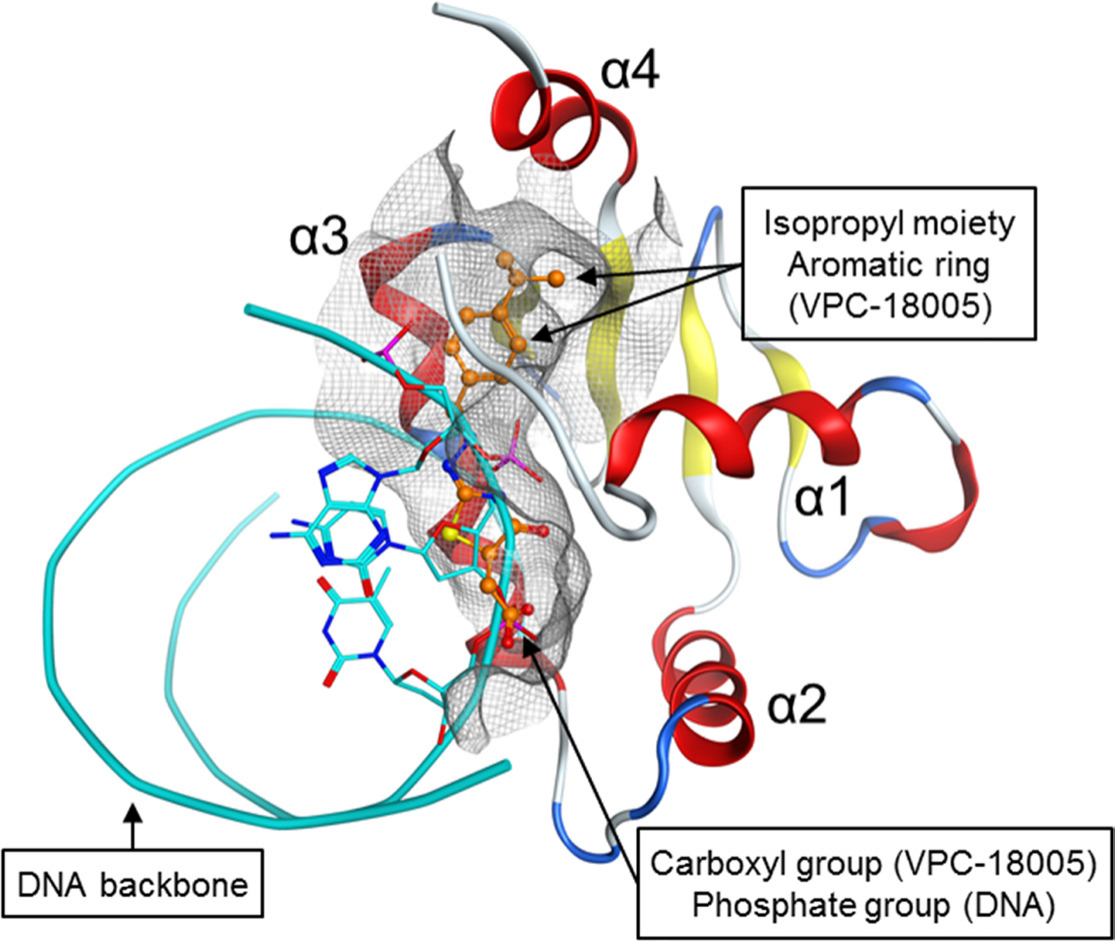
Mutually exclusive binding of VPC-18005 and DNA with the ERG-ETS domain The position of the carboxyl group in VPC-18005 (orange = carbon, blue = nitrogen, red = oxygen, yellow = sulfur) is predicted to coincide with that of a phosphate group on the DNA backbone (cyan ribbons and sticks). Thus, binding of VPC-18005 or DNA with the ERG-ETS domain is expected to be mutually exclusive. Whereas the lower portion of the VPC-18005 chemical structure occupies the same general area as the DNA, the upper portion, consisted of the isopropyl moiety and aromatic ring, extends further into the pocket (shown in grey net). The ERG-ETS protein structure is rotated −90 degree on the vertical axis, compared to those shown in Figure 2.

In contrast to these attempts, we utilized an established rational drug discovery approach [24] to directly target the DNA-binding interface of the ERG-ETS domain. NMR spectroscopy experiments demonstrated that VPC-18005 binds directly to the ETS domain of the ERG protein. The NMR data were consistent with the *in silico* modelling of the binding mode of VPC-18005 with ERG. In particular, the perturbation of Tyr371, a key residue required for the ERG-DNA interaction [25], by VPC-18005 observed in ^15^N-HSQC spectra provides a possible antagonizing mechanism. Superimposing VPC-18005 over the DNA at the ERG pocket further revealed the predicted mutually exclusive nature of their binding interfaces (Figure 6). Not only does VPC-18005 partially occupy the same interface as the DNA, but the negatively-charged carboxyl group is also mapped directly on top of the negatively-charged phosphate group of the DNA backbone.

It is also noteworthy that VPC-18005 binds the isolated ERG-ETS domain *in vitro* with mM affinity, and this is substantially weaker than the nM affinity interaction of ERG with its cognate DNA sequences. However, in the context of the cellular milieu, transcription factor binding to DNA is malleable and susceptible to chemical perturbations [42, 43]. Thus, VPC-18005 is biologically active when present in cell-based assays at μM concentrations. The differences between these *in vitro* versus *in vivo* results could arise for numerous reasons spanning from potentially elevated intracellular concentrations of VPC-18005 to highly sensitive effects of this compound on the network of cooperative intermolecular interactions required for transcription. Overall, VPC-18005 is the first reported small molecule inhibitor (SMI) that directly antagonizes the DNA-binding interface on the ERG protein, and it adds to the few successful examples where SMIs have been shown to disrupt protein-DNA interactions of transcription factors [42].

The use of a minimal promoter-Renilla luciferase control suggested that inhibition from VPC-18005 was specific to the ERG-responsive reporter and not a non-specific effect on general transcription (Supplementary Figure 9B). However, due to the sequence conservation at the ETS domain, it is expected that anti-ERG compounds such as VPC-18005 will have the potential to bind and inhibit other ETS factors. Indeed, preliminary NMR spectroscopic experiments revealed that VPC-18005 also interacts with the ETS domains of PU.1 and ETV4. Nevertheless, as demonstrated here, VPC-18005 is non-toxic at active concentrations and thus far has not demonstrated any specificity-related issues. It should also be noted that many of these ETS factors are oncogenic and have been implicated in a wide spectrum of cancers [4]. Although future development of new VPC-18005 derivatives can be prioritized based on their selective binding towards ERG, those showing promiscuous or increased specificities for alternative ETS factors should be considered as lead therapeutics towards cancers linked to those factors.

VPC-18005 inhibits ERG transcriptional activity in a dose dependent manner. Additionally, VPC-18005 can inhibit the expression of an ERG-regulated gene, *SOX9*, which has been previously shown to stimulate PCa invasion [29, 31]. VPC-18005 did not affect cell viability, but did influence cell motility, leading to reduced migration/invasion of cells *in vitro* and in a zebrafish xenograft model. As cancer cell death is a measure of toxicity and cancer cell immobility is a measure of metastasis prevention, our study supports non-toxic anti-metastatic applications of VPC-18005 and its derivatives. Future clinical studies of such anti-ERG drugs can be modelled based on previous clinical trials for anti-metastatic drugs to target patients with metastatic disease of low burden [44]. Key indicators of anti-metastatic drug efficacy in patients include inhibition of further metastasis/invasion of tissues, decreased skeletal related conditions, decreased pain/narcotic use, increased survival (decreased end organ destruction), and decrease of circulating tumor cells [45].

In summary, these results demonstrate proof-of-principal that small molecule targeting of the ERG-ETS domain can suppress transcriptional activity and reverse transformed characteristics of prostate cancers aberrantly expressing ERG. The current lead compound, VPC-18005, inhibited ERG with low micromolar concentrations at *in vitro* and *in vivo* experiments. In murine pharmacodynamics and toxicology studies, VPC-18005 is soluble, stable and orally bioavailable, and does not exhibit general toxicity at single doses of up to 500 mg/kg, and after a 4 week BID at 150 mg/kg trial (Supplementary Figure 10). We anticipate that future medicinal chemistry (medchem) efforts will improve its activity into a sub-micromolar or nanomolar range. Indeed, our initial medchem development has identified additional derivatives through chemical similarities and modifications of the VPC-18005 scaffold (Supplementary Table 3). Of these candidates VPC-18065 and 18098, with terminal moieties that are more hydrophobic, demonstrated slightly better IC_50_ values (2 μM and 1 μM respectively in luciferase assays) compared to VPC-18005 (Supplementary Figure 8). The removal of the carboxyl group in VPC-18100 resulted in the loss of inhibition in the luciferase reporter assays, as we expected given that the carboxylate is predicted to form a salt-bridge with the nearby Lys357. Although the modifications tested to date have not yet resulted in significant sub-micromolar activity, these derivatives do provide a working structure-activity relationship that will guide future medchem efforts. DNA binding domains of transcription factors are often conserved and exhibit low rates of mutations as a structural compromise is likely to translate into a loss of function [46]. Targeting such DNA-interacting regions may increase the value of the corresponding drugs due to less mutation-driven resistance, but also makes direct assessment of binding specificity by mutagenesis challenging.

PCa, one of the most common malignancies in men, is treated by surgery and radiation at the early stage, but eventually progresses to advanced forms that are managed primarily by the androgen deprivation therapy (ADT). The effectiveness of ADT is only temporary due to resistance mechanisms related to aberrant androgen production and mutations in the androgen receptor [47]. Recent studies not only established ERG as a critical drug target in PCa [7], but also reported on ERG feed-forward regulation. This supports the notion that despite initial dependence on the androgen receptor, ERG expression can eventually become self-driven and resistant against ADT [17]. Thus, anti-ERG drug prototypes such as VPC-18005 developed through rational drug design as reported here can specifically target the malignant transformation and metastasis driven by the ERG, and are not susceptible to current PCa treatment limitations such as drug resistance against anti-androgens and side effects from ADT. With the availability of non-invasive urine tests for ERG detection [9], future anti-ERG drugs can be specifically prescribed to the 50% of PCa patients who are ERG-positive and pave the way for precision medicine.

## MATERIALS AND METHODS

### *In silico* modeling and virtual screening

The published ERG-ETS domain X-ray crystal structure (PDB: 4IRG) [25] was subjected to the Site Finder algorithm, implemented in the Molecular Operating Environment (MOE) [48], which used virtual atomic probes to search the protein surface for suitable small molecule binding pockets. The crystal structure of an ERG/DNA complex (PDB: 4IRI) [25] was used to define the ERG-DNA interface. The top-ranked pocket was identified and used for the subsequent virtual screening. Before molecular docking, the ERG-ETS domain structural model was prepared by using the Protein Preparation Wizard module of the Maestro v9.3 program from the Schrodinger 2012 software suite. The docking grid was centered at the pocket composed of the following amino acids: Pro306, Gly307, Gln310, Ile311, Gln312, Leu313, Trp314, Trp351, Lys355, Met360, Lys364, Leu365, Ala368, Tyr371, Tyr372, Lys375, Ile377, Ile395, Ala398, Leu399 (residue numbering based on ERG isoform 5, UniProt ID: P11308-4). A total of 19,607,722 (~ 20 million) small molecule structures were downloaded from the ZINC database version 12 [26]. Among the 20 million set, a total of 2,990,102 (~ 3 million) molecules that possess the following lead-like and drug-like properties were extracted for molecular docking: molecular weight between 250 and 400 Da, logP <= 5, hydrogen-bond donors <= 5, hydrogen-bond acceptors <= 10, number of rotatable bonds <= 10, and number of rings <= 4. Each molecule was given its expected protonation state at pH 7 and energy-minimized under the MMFF94x (solvation: Born) force field using MOE. Each molecule was docked into the previously defined docking grid on the ERG-ETS domain protein model, using the Glide program (Small-Molecule Drug Discovery Suite, version 5.8, Schrödinger, LLC, New York, NY, 2012). Standard Precision with all other parameters set to default. The top 1% (~30,000 molecules), as ranked by the docking scores calculated based on interaction forces including hydrogen bonds and hydrophobic interactions, were selected to advance into the next stage of virtual screening. Within this set, a predicted pK_i_ was calculated for each molecule using a custom MOE SVL script, and ligand efficiency was calculated using Glide. In addition, this set of 30,000 molecules was re-docked into the same pocket, using the eHiTs docking program [49]. A root-mean-square deviation (RMSD) was calculated between the docking poses from Glide and eHiTs for each molecule. A consensus scoring (voting) method was used each compound received one vote from each of the following criteria met: 1) top 20% pK_i_ values, 2) top 20% ligand efficiency values, and 3) top 20% eHiTs docking scores and 4) RMSD <=3 A. The top 3,000 molecules, as ranked by the number of votes, were selected for the final stage of selection. During this step, the chemical structure of each molecule within the predicted ERG-ETS binding pocket was manually examined using the 3D visual environment in MOE. Preference was given to compounds with favorable binding poses and interactions with the surrounding amino acid residues. Molecules were removed from the selection if they contain problematic or promiscuous moieties. In addition to manual examination, the FAFDrugs program [50] was used to assist identification of such problematic groups. A total of 48 compounds were selected for testing. MOE and MarvinSketch were used to visualize and represent the protein models and chemical structures. Chemical similarity searches based on the Tanimoto coefficient was performed on the hit compound VPC-18005 (prepared as detailed in Supplementary Materials and Methods) in the ZINC database, with additional medchem derivatives designed using MOE.

### Cell culture

VCaP (CRL-2876) and PC3 (CRL-1435) human prostate carcinoma cells were obtained from the American Type Culture Collection (ATCC). VCaP cells harbor an endogenous *TMPRSS2-ERG* gene fusion, whereas PC3 cells do not, but do express the ETS family member ETV4. The immortalized prostatic epithelial cell line, PNT1B [27] was purchased from ATCC. PNT1B-Mock, PNT1B-ERG, RWPE-Mock, and RWPE-ERG cells are lineage-matched control and ERG-expressing prostatic epithelial lines generated in house [14]. PC3 cells were maintained in RPMI 1640 medium (Life Technologies) supplemented with 5% (v/v) fetal bovine serum (FBS). VCaP cells were maintained in low bicarbonate DMEM (ATCC) supplemented with 10% FBS. PNT1B-Mock and –ERG cells were maintained in DMEM (Life Technologies) supplemented with 10% FBS and under blasticidin selection. Cells were grown in a humidified, 5% CO_2_ incubator at 37^°^C.

### Western blot

Cells were lysed on ice with RIPA buffer containing a protease inhibitor cocktail (Pierce). Primary antibodies: ERG (1:1,000, EPR3864(2), Abcam), α-Tubulin (1:20,000, Millipore), Vinculin (1:1,000, Abcam). Immunoreactivity was detected with the use of the goat anti-rabbit or rabbit anti-mouse horseradish peroxidase (HPR)–conjugated secondary antibody (1:10,000) (Santa Cruz), and visualization was achieved by chemiluminescence (Pierce). To inhibit protein synthesis, 10 μM cycloheximide was added for 1 h and then replaced with treatment medium for indicated time frame.

### Dual reporter luciferase assay

All of the compounds selected from the virtual screening were tested in a luciferase-based ERG-responsive reporter assay, using two ERG-overexpressing cell lines, VCaP and PNT1B-ERG, previously developed in house [14]. Cells (3000) in 150 μL per well of a 96 well plate were seeded and after a 24 h incubation were transfected with 50 ng of an Endoglin E3 promoter-derived ETS-responsive firefly luciferase reporter (−507/−280 of (E3) promoter [28] inserted into luciferase reporter vector (Signosis), ARR_3_tk-luc [24], and 5 ng of the Renilla luciferase reporter (pRL-tk, Promega) using TransIT 20/20 transfection reagent (Mirus, USA). After 16 h incubation, treatment media was added for further 48 h. Firefly and Renilla luciferase activities were measured using a TECAN M200Pro plate reader. Comparison of empty vector versus ETS responsive reporter demonstrates activation only in the presence of the ETS responsive sequence (Supplementary Figure 9A). Data were normalized first to Renilla luciferase and then to the DMSO-media control on each plate, unless otherwise stated. Initial hit compounds were identified as those with an average normalized luciferase reading (firefly luciferase/Renilla luciferase readings) that is 60% or less of the average normalized luciferase reading of the DMSO-media control (i.e. 40% or more reduction of luciferase activity) at 10 μM. Representative example of raw Renilla luciferase readings presented in Supplementary Figure 9B. The luciferase assays were repeated for each lead compound under multiple concentrations (0.1 to 100 μM) to establish a dose-dependent response and an IC_50_ value. AR reporter assay was performed as previously described [24].

### Proliferation/ cell viability assay

3-(4,5-dimethylthiazol-2-yl)-5-(3-carboxymethoxyp henyl)-2-(4-sulfophenyl)-2H-tetrazolium, inner salt (MTS). Cells were seeded at a density of 3000 cells per well (except VCaP at 20,000/well) in 100 μL of appropriate media in 96 well culture dishes. Twenty four hours later, 100 μL of medium containing vehicle control or compounds. Each treatment was prepared in triplicate. After a 72 h treatment, cellular viability was assessed using CellTiter 96 ^®^ Aqueous One Solution Cell Proliferation Assay reagent (Promega) according to the manufacturer's instructions. Values were normalized to the DMSO control.

### NMR spectroscopy

ERG-ETS domain expression and purification. A pET28a plasmid encoding residues 307–400 of the ERG-ETS domain was expressed in *E. coli BL21 (λDE3)*. Cultures of 1 L were grown at 37°C in M9 media supplied with 3 gm/L ^13^C_6_-glucose and/or 1 gm/L ^15^NH_4_Cl. Cells were allowed to grow to O.D._600_ = 0.6 and protein expression was induced by adding 1 mM IPTG. After an induction time of 4 h, cells were harvested by centrifugation and stored at −80^°^C for at least 1 round of freeze/thaw. Cells were resuspended in 40 mL of lysis buffer for every 1 L of culture. Cells were lysed by passing through 5 rounds of homogenization and 10 mins of sonication. The cell lysate centrifuged at 15k rpm for 1 hr, and the supernatant subjected to nickel column purification. The column was washed using 25 mM imidazole (50 mM phosphate, 1 M NaCl, pH 7.4) and proteins were eluted with 1 M imidazole. Fractions containing the ETS domain were confirmed by SDS-PAGE and pooled. The His_6_-tag was cleaved by thrombin and the tag-free sample was concentrated to 2 mL and subjected to S75 size exclusion chromatography. Fractions were checked by SDS-PAGE and those containing the pure sample were pooled and concentrated. The protein ample was dialyzed to NMR buffer (20 mM sodium phosphate, 150 mM NaCl, 2 mM DTT, 0.1 mM EDTA, pH 6.5) for all NMR experiments. NMR spectral assignments. NMR data were recorded at 25 or 28°C on cryoprobe-equipped 850 MHz Bruker Avance III spectrometer. Data were processed and analyzed using NMRpipe [51] and Sparky [52]. Signals from backbone and sidechain ^1^H, ^13^C, and ^15^N nuclei were assigned by standard multidimensional heteronuclear correlation experiments. NMR-monitored titrations. Interactions of compounds with the ERG-ETS domain were monitored via sensitivity-enhanced ^15^N-HSQC spectra. Experiments involved titrating unlabeled DMSO-solubilized compound or control DMSO into ^15^N-labeled ERG-ETS domain. Chemical shift perturbations were calculated from the combined amide ^1^H^N^ and ^15^N shift changes as Δδ = [(0.2 × Δδ_N_)^2^ + (Δδ_H_)^2^]^1/2^. Reciprocal titrations were carried out using ^1^H-NMR to monitor the effects of progressively adding unlabeled protein to a sample of VPC-18005 (180 μM) in 20 mM phosphate, 150 mM NaCl, 2 mM DTT, 0.1 mM EDTA, pH 6.5. The signal from water was suppressed by pre-saturation.

### Electrophoretic mobility shift assay (EMSA)

Purified ERG-ETS domain (see NMR spectroscopy) was stored in buffer (20 mM sodium phosphate, 200 mM NaCl, 2 mM DTT, 0.1 mM EDTA, pH 6.5). To prepare the probe for the gel shift assay, equal amounts (200 nM) of Alexa-488 fluorophore-labeled DNAs (5′-CGGCC AAGCCGGAAGTGAGTG-3′ and its complement) were mixed, heated to 95°C for 30 minutes, and then slowly cooled to 25°C in several hours. An initial gel shift assay was performed by titrating constant 1 nM labeled dsDNA with ERG at concentrations spanning 0.3 pM to 0.5 μM. Glycerol (3%) and 0.2 mg/mL bovine serum albumin (BSA) were included in the reaction mixture. After incubated at room temperature for 1 hr, samples were load on to 10% polyacrylamide native gel, and electrophoresed at 10^°^C. The gel was scanned with Typhoon 9200 Imager equipped with blue laser to excite at 490 nm and fluorescence was measured at 520 nm. The scanned image was analyzed with Image J [48]. Non-linear least squares fitting (GraphPad Prism) of the titration data to a 1:1 binding isotherm yielded the equilibrium dissociation constant (K_D_ value ~ 1 nM) for the ERG- ETS domain interaction with DNA. The binding isotherm equation is f_b,i_ = [ERG]_i_/([ERG]_i_ + K_D_) where [ERG]_i_ is the total concentration of the ERG-ETS domain (a valid approximation as K_D_ > 1 nM total dsDNA) at each titration point (i), and the fraction bound, f_b,i_ was calculated as the intensity of the bound DNA band at that point relative to the intensity with saturating 0.5 μM protein. The result of this initial study was used to set the molar ratio of ERG-ETS domain:DNA in subsequent competition assays with VPC-18005. For these assays, 4 nM of the ERG-ETS domain was mixed with 1 nM of fluorophore-labeled dsDNA, titrated with VPC-18005 (diluted from a DMSO stock) and analyzed by the same EMSA protocol. The data were fit to the equation for competitive binding, f_b,i_ = [ERG]/([ERG] + K_D_{1 + [VPC-18005]_i_/K_I_}), where K_I_ is the inhibitor dissociation constant and the fraction bound, f_b,i_, was calculated the intensity of the bound DNA band at each titration point relative to that without added VPC-18005. A control experiment was carried out by titrating with equivalent quantities of DMSO.

For experiments involving ERG from VCaP cells, VCaP nuclear protein was extracted using CelLytic NuCLEAR Extraction Kit (Sigma). An initial gel shift assay was performed by titrating constant 1 nM labeled dsDNA nuclear extract at concentrations spanning 1.1 pg/μl to 1.76 μg/μl (Supplementary Figure 6C). For subsequent assays, 55 ng/μl of the nuclear extract was mixed with 1 nM of fluorophore-labeled dsDNA, titrated with VPC-18005 (diluted from a DMSO stock) and analyzed by the same EMSA protocol.

### Analyses of gene expression

Total RNA was extracted from VCaP cells with the use of RNeasy Plus kit (Qiagen). Reverse transcription was performed with the use of the iScript First-Strand cDNA Synthesis Kit (Bio-Rad Laboratories) with 100 ng total RNA used as template. Real time reverse-transcription (RT) polymerase chain reaction (PCR) primers for ERG synthesized by IDT (forward, 5′- CGCAGATTATCGT GCCAGCAGAT -3′; reverse, 5′- CCATATTCTTTCACC GCCCACTCC-3′) and SOX9 (Quantitect primer assay, Qiagen). Real-time quantitative RT-PCR was performed in triplicate for each sample with the use of the ABI ViiA7 QPCR thermocycler. In each reaction, 1 μl cDNA, 1 μl forward and reverse primers (or 1 μl of Quantitect primers), and 6 μl Sybr Green Master Mix (Applied Biosystems) were added with water to make a final volume of 12 μl. All primers were used at a concentration of 5 μmol/l. PCR cycling conditions were 95°C for 10 minutes followed by 40 cycles of 95°C for 15 seconds, 60°C for 1 min. Data was normalized to reference genes: GAPDH (forward, 5′- CCATATTCTTTCACCGCCCACTCC -3′; reverse, 5′- GGCATGGACTGTGGTCATGAG -3′) The *2*^−ΔΔ^*CT* method was used to compare samples. PCR product specificity was validated with the use of a melt curve.

### Real time cell analysis (xCELLigence)

Cell migration was monitored using CIM-16 migration plates via the xCELLigence platform (ACEA). FBS-supplemented media (160 μL) was added to the lower chamber of the plate and incubated at RT for 30 min. The upper chamber was then mounted and 30 μL of serum free media (SFM) was added to each well and left to equilibrate in the incubator for 1 h at 37°C. After the incubation, a background reading was taken for each well. PNT1B-ERG or –MOCK cells, cultured for 24 h in SFM, were seeded into the wells of the upper chamber at 30,000 cells per well and after 24 h 100 μL of desired treatment was added (vehicle control, VPC-18005, and YK-4-279). Real time readings of cell index values were recorded initially every 5 min until the end of the experiment (48 hr).

### Spheroid invasion assay

3D Spheroid BME Cell Invasion Assay (Trevigen) was performed as per manufacturer's instructions. Briefly, 5,000 PNT1B-ERG cells and 5 μL of ECM were prepared in growth media to a total volume of 50 μL and seeded in 3D culture qualified 96 well spheroid formation plate and incubated at 37°C for 72 hr. Spheroids were pre-treated with VPC-18005 or DMSO for 24 h after which 50 μL gel invasion matrix was added. Spheroids were then incubated at 37°C for 3 to 7 days, and photographed using Zeiss AxioObserver Z1 microscope in each well on the day of invasion mix addition and every two days following. Spheroids were retreated with 50 μL of vehicle control or compound after 72 hr.

### Zebrafish

Research was carried in accordance with protocols compliant to the Canadian Council on Animal Care and with the approval of the Animal Care Committee at the University of British Columbia. The wildtype zebrafish strain was maintained in aquaria according to standard protocols [53]. Embryos were generated by natural pair-wise matings and raised at 28.5°C on a 14 h light/10 h dark cycle in a 100 mm^2^ petri dish containing aquarium water. Phenylthiourea (0.2 mM PTU, Sigma) was added to the embryos at 10 h post-fertilization (hpf) to prevent pigment formation. Yolk sac dissemination assay. PCa cell lines were fluorescently labelled the day before microinjection with 1.5 μM of CellTracker CM-Dil dye (Life Technologies) as per manufacturer's instructions. Wild-type embryos were dechorionated at 2 dpf. Following anaesthetization with tricane, approximately 50–70 cancer cells were microinjected into the yolk sac. Embryos were then transferred to 100 mm^2^ plates that contained aquaria water with added PTU and VPC-18005, YK-4-279 or DMSO control. Embryos were visually assessed for presence of xenograph. Those embryos that did not contain cells were removed from the experiment. Embryos were kept at 35°C for the duration of the experiment. Approximately, 50 fish were injected per cell line and metastasis was determined on Day 4 and 5 by observation using the Zeiss Axio Observer microscope (5X objective) controlled with Zen 2012 software. Fixed (dead) cells were used as a control to ensure that the dissemination observed was not due to yolk sac absorption.

### Statistics

Data are presented as mean ± standard error of the mean (SEM) unless indicated otherwise. The Kruskal–Wallis test with Dunn's Multiple Comparison post hoc test, chi squared test, two-way ANOVA followed by Fisher's LSD post hoc test, and *t*-test were used for analyses as indicated in the respective figure legends. *p* < 0.05 was considered significant. Statistical analyses were performed with the use of GraphPad Instat or GraphPad Prism 6 (GraphPad Software, Inc.).

## SUPPLEMENTARY VIDEO, TABLES and FIGURES






