# Supplementary material for: Discovery and characterization of small molecules targeting the DNA-binding ETS domain of ERG in prostate cancer
Source: Oncotarget. 2017 Apr 15;8(26):42438–54. doi: 10.18632/oncotarget.17124 (PMC5522078; doi:10.18632/oncotarget.17124)
Supplement: Supplementary file 1 [file oncotarget-08-42438-s001.pdf]

Discovery and characterization of small molecules targeting the DNA-binding ETS domain of ERG in prostate cancer

Supplementary Materials

SUPPLEMENTARY MATERIALS AND METHODS

Compound solubility and stability

Stock solutions of compounds at 50 mM in dimethyl sulfoxide (DMSO) were diluted 1,000× into methanol (MeOH), RPMI + 5% charcoal stripped serum (CSS) (media), and phosphate buffered saline (PBS) and vortex mixed for 1 hr, 800 rpm at room temperature (RT). The resulting solutions were centrifuged at 20,000 g for 5 min (RT) and saturated supernatants were transferred to fresh Eppendorf tubes. Saturated PBS samples were further diluted with an equal volume of PBS. Aliquots of these solutions were analysed and the remainder stored at RT in the dark. Aliquots taken at later time points were vortex mixed for 1 h prior to sampling. MeOH and diluted PBS samples required no further processing; media samples were extracted with two volumes acetonitrile (ACN) and centrifuged at 20,000 g for 5 min. These MeOH, and diluted media and PBS samples, were analysed using an Acquity UPLC coupled in series with an eLambda PDA and a Quattro Premier (Waters). A 100 mm BEH C18, 1.7 µ column (Waters) was used for separations with a 10–95% acetonitrile (ACN) gradient from 0.2–7 min followed by a 1 min 95% ACN flush and 2 min re-equilibration for a 10 min run length (0.1% formic acid present throughout). Wavelengths from 210–800 nm at 1.2 nm resolution and 2 points/sec were collected with the PDA. The sampler was maintained at RT and all MS data was collected in ES+ scan or single ion recording (SIR) mode at unit resolution with the following instrument parameters: capillary, 3.0 kV; extractor and RF lens, 3 V and 0.1 V; cone, 40 V; source and desolvation temperatures, 120°C and 350°C; desolvation and cone (N2) flow, 900 L/hr and 50 L/hr. The m/z for SIR functions were selected from MeOH scan datasets.

Quanlynx (Waters) was used for analysis of data, using extracted wavelength chromatograms selected for best signal to noise for PDA data and SIR for MS data. All compounds dissolved well in MeOH and these were used for calibration purposes with slopes forced through the origin. OD data was used in most cases with MS data mainly for PBS samples; SIR data was calibrated by applying the SIR/OD ratio from corresponding media samples where less saturation of MS data is expected. This rudimentary method is useful to 50 µM, performs well for solubility and relative stability at higher concentrations and gives reasonable estimates when the use of MS endpoints is needed.

Cell cycle analysis

Cells were detached by treatment with Accutase (Gibco), then underwent APC BrdU Flow Kit protocol (BD Pharmingen). Cells were analysed on a FACSCanto™ II (BD Biosciences). Data was analyzed using FlowJo software (TreeStar, USA). Biological replicates were analysed statistically by Two-Way ANOVA.

Proliferation/cell viability assay

Incucyte generated growth curves

VCaP cells (20,000 cells/well) were plated in a 96 well plate. After 24 h, plates were treated with vehicle control, VPC-18005 or YK-4-279 at the indicated concentrations. Growth curves were constructed by imaging plates using the Incucyte system (Essen Instruments), where the growth curves were built from real-time confluence measurements acquired during round-the-clock kinetic imaging for 7 days.

Bioinformatics and statistical analyses on gene expression datasets from PCa patients

The gene expression datasets included 26 PCa and 5 normal patient samples from Vancouver Prostate Centre (VPC) [1], 150 PCa and 29 normal patient samples from Memorial Sloan-Kettering Cancer Center (MSKCC) [2], and 498 PCa and 52 normal patient samples from The Cancer Genome Atlas (TCGA) [3]. A list of upregulated genes were identified from each dataset by the following steps: 1) log2 transformation; 2) two sample t-test between tumor and normal samples; 3) multiple testing correction on *p*-values; 4) selection of genes with corrected (adjusted) *p*-values < 0.05; and 5) among those with significant *p*-values, selection of genes with fold-change ≥ 2 (tumor vs. normal).

Chemical synthesis of VPC-18005 (Supplementary Figure 9C)

General experimental procedures

All reagents and solvents were purchased from commercial suppliers and used without further purification unless otherwise stated. The reactions were monitored by thin layer chromatography (TLC) on pre-coated silica gel F254 plates (Sigma-Aldrich) with a UV indicator using ethylacetate/hexane (1:2 v/v). Yields were of purified product were not optimized. The purities of the newly synthesized compounds were determined by LC-MS analysis using an Agilent 1100 LC system. The compound solution was injected into the ionization source operating positive and negative modes with a mobile phase acetonitrile/water/formic acid (50:50:0.1% v/v) at 1.0 mL/min. The instrument was externally calibrated for the mass range m/z 100 to 650. The ^{1}H-NMR spectra were measured on a Varian GEMINI 2000 NMR spectrometer system with working frequency of 400 MHz. Chemical shifts δ are given in ppm, and the following abbreviations are used: singlet (s), doublet (d), triplet (t), quartet (q), multiplet (m), and broad singlet (br s). VPC-18005: 2-((Z)-2-(((Z)-4-isopropylbenzylidene) hydrazono)-4-oxothiazolidin-5-yl)acetic acid (4d). To a stirred solution of 4-isopropylbenzaldehyde (1d) (563 mg, 3.8 mmol) in PhMe (2 mL) and DMF (2 mL) were added thiosemicarbazide (2) (290 mg, 3.2 mmol) and p-TsOH acid (5 mg, 0.03 mmol). The reaction mixture was heated in stirred microwave vial for 10 min at 90°C. After formation of thiosemicarbazone derivate and testing by TLC, maleic anhydride (3) (343 mg, 3.5 mmol) was added, and the reaction mixture was heated for 40 min at 110°C in the microwave. Recrystallized from AcOH yielded (4d, VPC-18005) (300 mg, 29% yield) as a white solid with 99% purity by LC/MS. ^{1}H-NMR (DMSO-d_6, 400 MHz): 1.20–1.22 (6H, d), 2.69–2.76 (1H, m), 2.90–2.94 (2H, m), 4.25–4.28 (1H, d), 7.31–7.33 (2H, d), 7.66–7.68 (2H, d), and 8.34 (1H,s). MS (ESI) m/z $(M + H)^+$ calculated for $C_{15}H_{17}N_3O_3S$: 319.4, found: 320.2. The final product is racemic and has several possible isomeric forms that have not been experimentally defined.

REFERENCES

1. Wyatt AW, Mo F, Wang K, McConeghy B, Brahmbhatt S, Jong L, Mitchell DM, Johnston RL, Haegert A, Li E, Liew J, Yeung J, Shrestha R, et al. Heterogeneity in the inter-tumor transcriptome of high risk prostate cancer. Genome biology. 2014; 15:426.
2. Taylor BS, Schultz N, Hieronymus H, Gopalan A, Xiao Y, Carver BS, Arora VK, Kaushik P, Cerami E, Reva B, Antipin Y, Mitsiades N, Landers T, et al. Integrative genomic profiling of human prostate cancer. Cancer cell. 2010; 18:11–22.
3. The Molecular Taxonomy of Primary Prostate Cancer. Cell. 2015; 163:1011–1025.

Supplementary Video 1: – Z-stack microscopy of zebrafish dissemination model. PNT1B-ERG cells were microinjected into the yolk sac of the zebrafish and the metastasis capability of the cells were detected using confocal microscope. Five days following injection, ERG expressing cells had invaded and metastasized into the head and tail region of the fish. See Supplementary_Video_1

Supplementary Table 1: Overexpressed genes common in the VPC and TCGA gene expression sets. See Supplementary_Table_1

Supplementary Table 2: Overexpressed genes common in the MSKCC and TCGA gene expression sets

Gene Symbol	NCBI Gene ID	*P*-value (MSKCC)	Adjusted P-value (MSKCC)	Fold change (MSKCC)	*P*-value (TCGA)	Adjusted *P*-value (TCGA)	Fold change (TCGA)
ABCC4	10257	8.29E-12	6.78E-10	2.06	3.56E-11	2.58E-10	3.62
ACSM1	116285	6.75E-20	9.77E-17	2.72	3.91E-14	5.30E-13	7.13
AMACR	23600	1.57E-22	7.58E-19	4.03	3.76E-27	1.31E-24	8.17
ARHGEF38	54848	6.68E-12	5.70E-10	2.39	5.11E-11	3.59E-10	4.10
CRISP3	10321	2.36E-11	1.75E-09	2.30	2.49E-02	4.05E-02	2.72
DNAH5	1767	4.22E-14	7.77E-12	2.48	6.80E-15	1.10E-13	5.66
EPCAM	4072	6.18E-12	5.31E-10	2.02	3.32E-14	4.57E-13	2.27
ERG	2078	7.77E-18	3.75E-15	2.66	6.60E-11	4.53E-10	2.90
GCNT1	2650	6.01E-13	7.43E-11	2.02	3.31E-14	4.57E-13	3.18
GLYATL1	92292	9.86E-13	1.13E-10	2.29	4.00E-09	1.99E-08	5.88
OR51E1	143503	6.83E-06	8.76E-05	2.01	5.53E-04	1.20E-03	3.01
PCA3	50652	2.11E-16	7.01E-14	4.84	6.46E-15	1.05E-13	28.21
SIM2	6493	1.11E-10	6.44E-09	3.02	5.32E-16	1.12E-14	7.94
TARP	445347	9.56E-06	1.16E-04	2.23	4.92E-10	2.89E-09	7.06
TDRD1	56165	8.69E-30	3.77E-25	3.05	1.72E-18	6.92E-17	17.90
TMEFF2	23671	4.74E-05	4.45E-04	2.06	6.16E-04	1.33E-03	2.24
TRIB1	10221	3.35E-11	2.35E-09	2.31	2.99E-12	2.67E-11	2.67

Supplementary Table 3: A list of active VPC-18005 derivatives

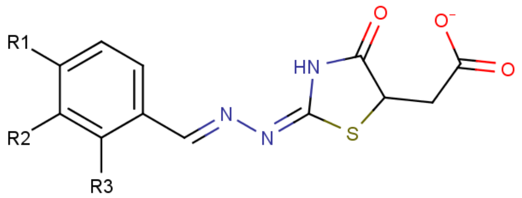

VPC-ID	R1	R2	R3	IC50 in Luciferase assay PNT1B-ERG cells	Solubility in media
18005	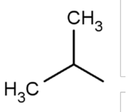	**H**	**H**	3 µM	> 50 µM
18065	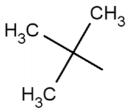	**H**	**H**	2 µM	> 50 µM
18098	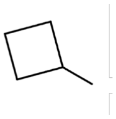	**H**	**H**	1 µM	> 50 µM
18104	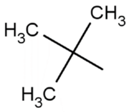	**H**	**OH**	3 µM	n.d.
18106	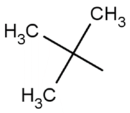	**F**	**H**	4 µM	n.d.
18113	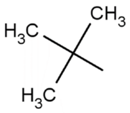	**Cl**	**H**	8 µM	n.d.
18114	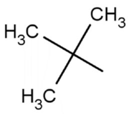	**H**	**F**	8 µM	n.d.
18118	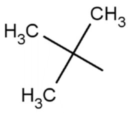	**H**	**CH3**	2 µM	n.d.
18119	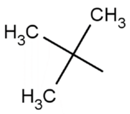	**CH3**	**H**	4 µM	n.d.
18120	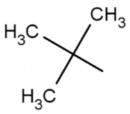	**H**	**Cl**	1 µM	n.d.

n.d. = not determined.

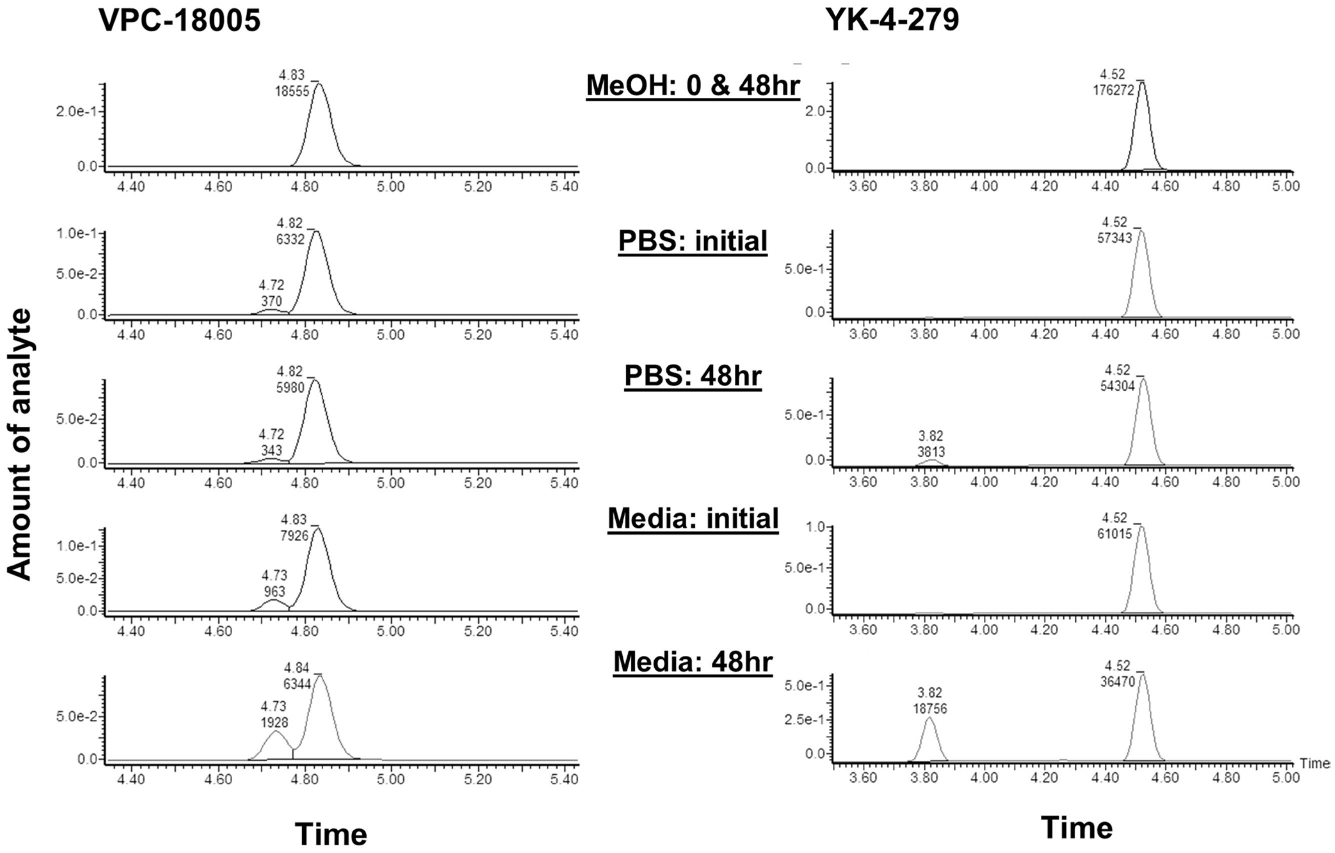

Supplementary Figure 1: VPC-18005 is stable and soluble. Solubility of VPC-18005 was assessed relative to YK-4-279 by diluting stocks (50 mM in DMSO) 1000x in Methanol (standard), PBS, or media. Resulting solutions were clarified by centrifugation and an aliquot of the supernatant was extracted with 2 volumes acetonitrile. VPC-18005 solubility determined as fraction remaining as quantified by LC-PDA-MS versus the methanol solution as standard.

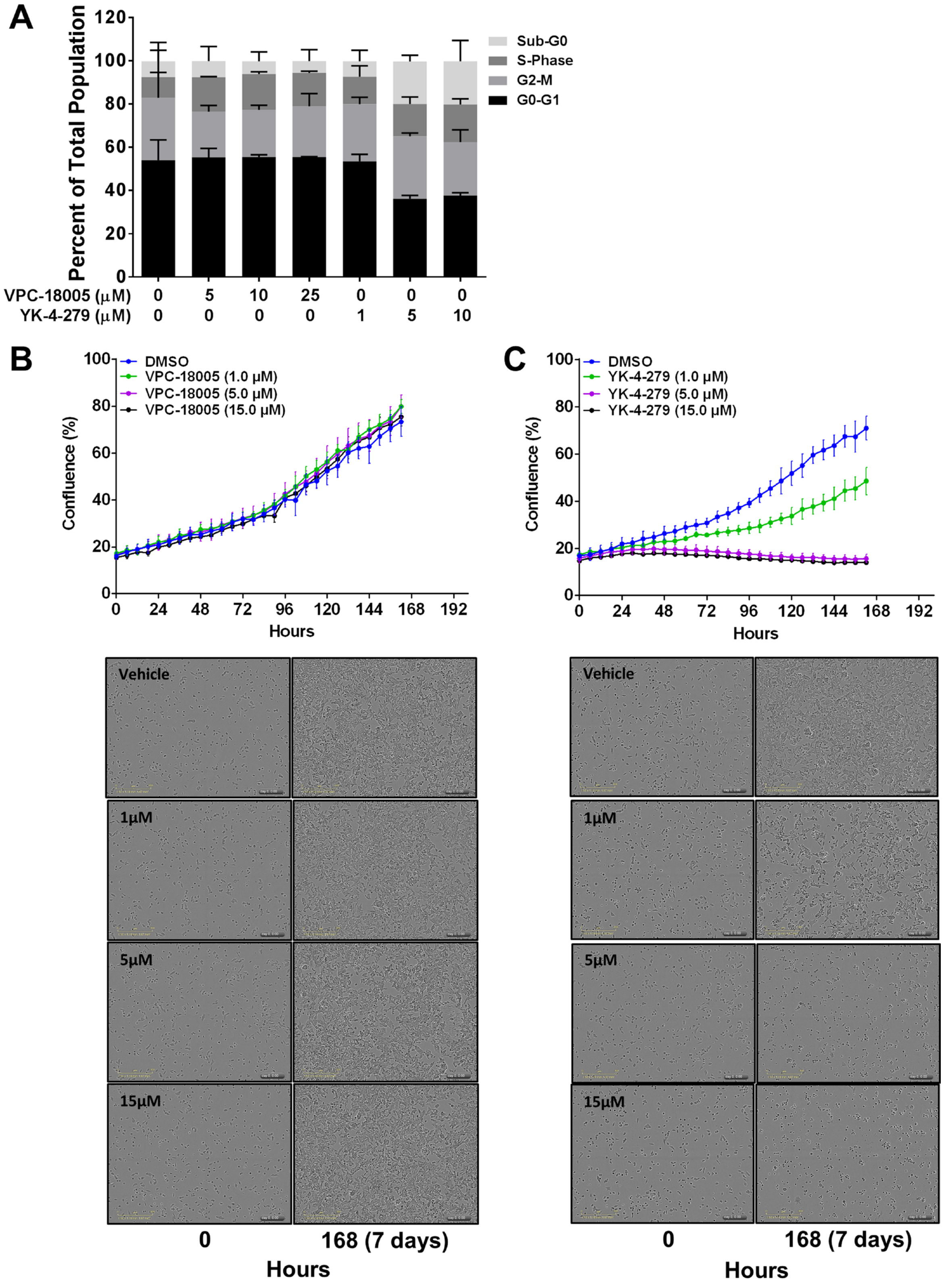

Supplementary Figure 2: *In vitro* assessment of YK-4-279 and VPC-18005 on cell proliferation. (**A**) Cell cycle analysis of VCaP cells treated with VPC-18005 and YK-4-279 was performed by flow cytometyry analysis of fixed, BrDU stained cells. Gates for sub G_0, G_0–G_1, S-phase and G_2-M populations were established for untreated cells and applied to all treated cell populations. Histograms represent the mean ± SEM of triplicate experiments. Significant differences were deduced by two-way ANOVA followed by Fisher›s LSD post hoc test. The sub-G_0 population was significant at 5 and 10 μM YK-4-279 doses (p = 0.003 and 0.003, respectively). The G_0–G_1 phase population is significant at 5 and 10 μM YK-4-279 treatment (p = 0.003 and 0.006, respectively). Growth assay of PNT1B-ERG cells treated with (**B**) VPC-18005 or C) YK-4-279. Cells were grown on cell culture plates and treated with vehicle (DMSO, blue) or compounds at 1, 5, or 15 μM (green, purple, black, respectively). The confluency of the wells (indicated by the lower panels) was measured by the live-cell imaging system IncuCyte™ (Essen Instruments) for 168 h (7 days).

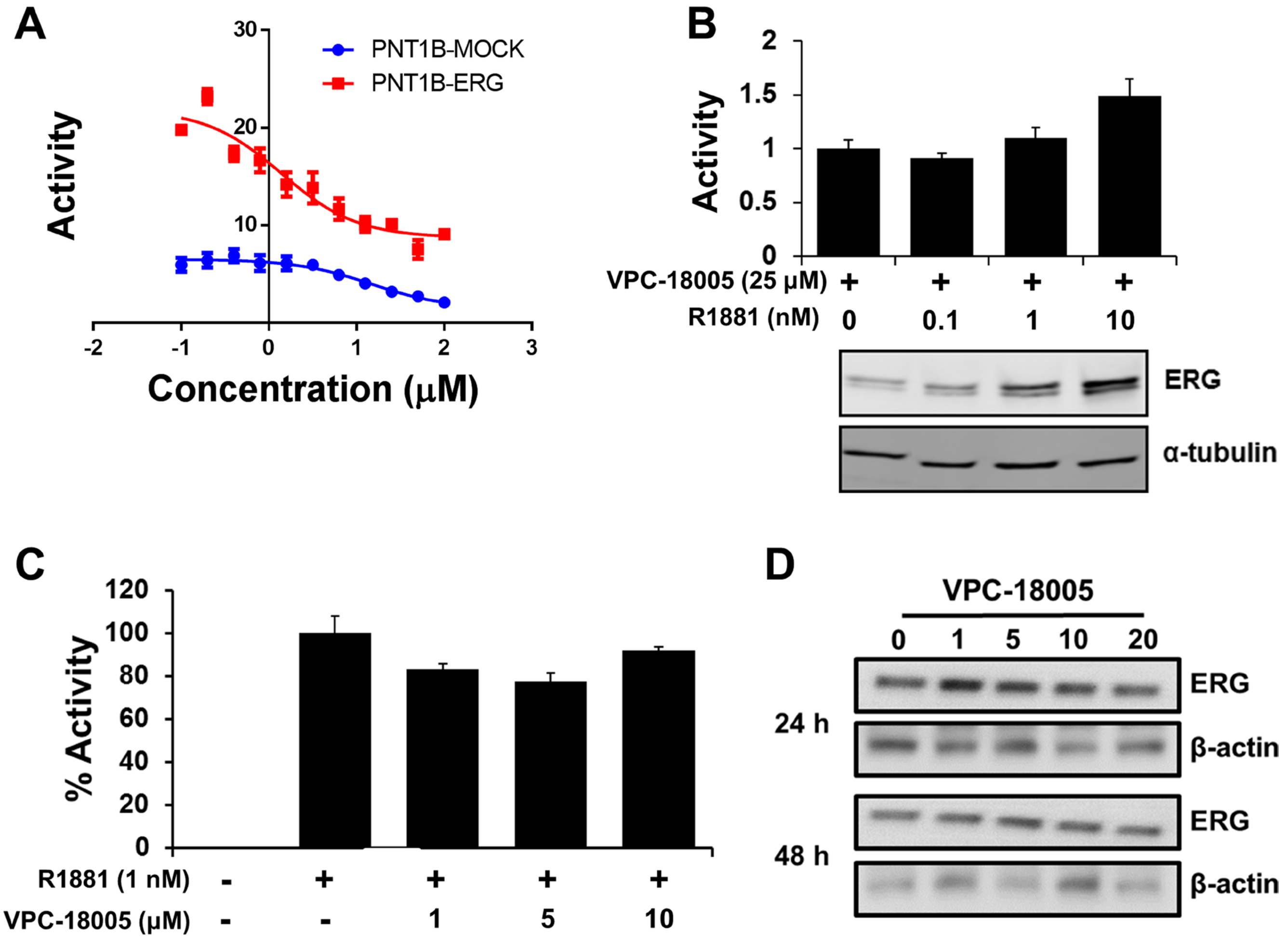

Supplementary Figure 3: VPC-18005 selectively suppresses ERG-mediated transcriptional activity. (**A**) PNT1B-ERG (red) and –MOCK (blue) cells were transfected with ETS responsive luciferase reporter and treated with VPC-18005 at the indicated concentration for 48 h. Data are presented as the mean ± SEM of 4 technical replicates and expressed as luciferase:renilla ratio (activity). (**B**) Luciferase assay was performed on VCaP cells transfected with ETS responsive luciferase reporter and treated with DMSO (0) or 0.1, 1, or 10 nM R1881 in the presence of 25 μM of VPC-18005. Data are presented as the mean ± SEM of 4 technical replicates and expressed as the luciferase:renilla ratio relative to VPC-18005 alone. Lysates from harvested cells were immunoblotted for ERG (upper panel) or α-tubulin (α-tubulin; lower panel) as a loading control. (**C**) PC3 cells were transfected with AR and the androgen-responsive promoter, ARR3TK-Luc. Cells were treated with DMSO vehicle (0), or 1 nM synthetic androgen (R1881) ± VPC-18005 at 1, 5 or 10 μM, Data are presented as the mean ± SEM of 4 technical replicates and expressed as the percentage luciferase expression (% Activity) relative to R1881. (**D**) VCaP cells were treated for 1 h with cycloheximide (10 μM) then cultured for 24 and 48 h with VPC-18005 at the indicatedμM concentrations. Lysates were immunoblotted for ERG (upper panels) and b-actin (lower panels) as a loading control.

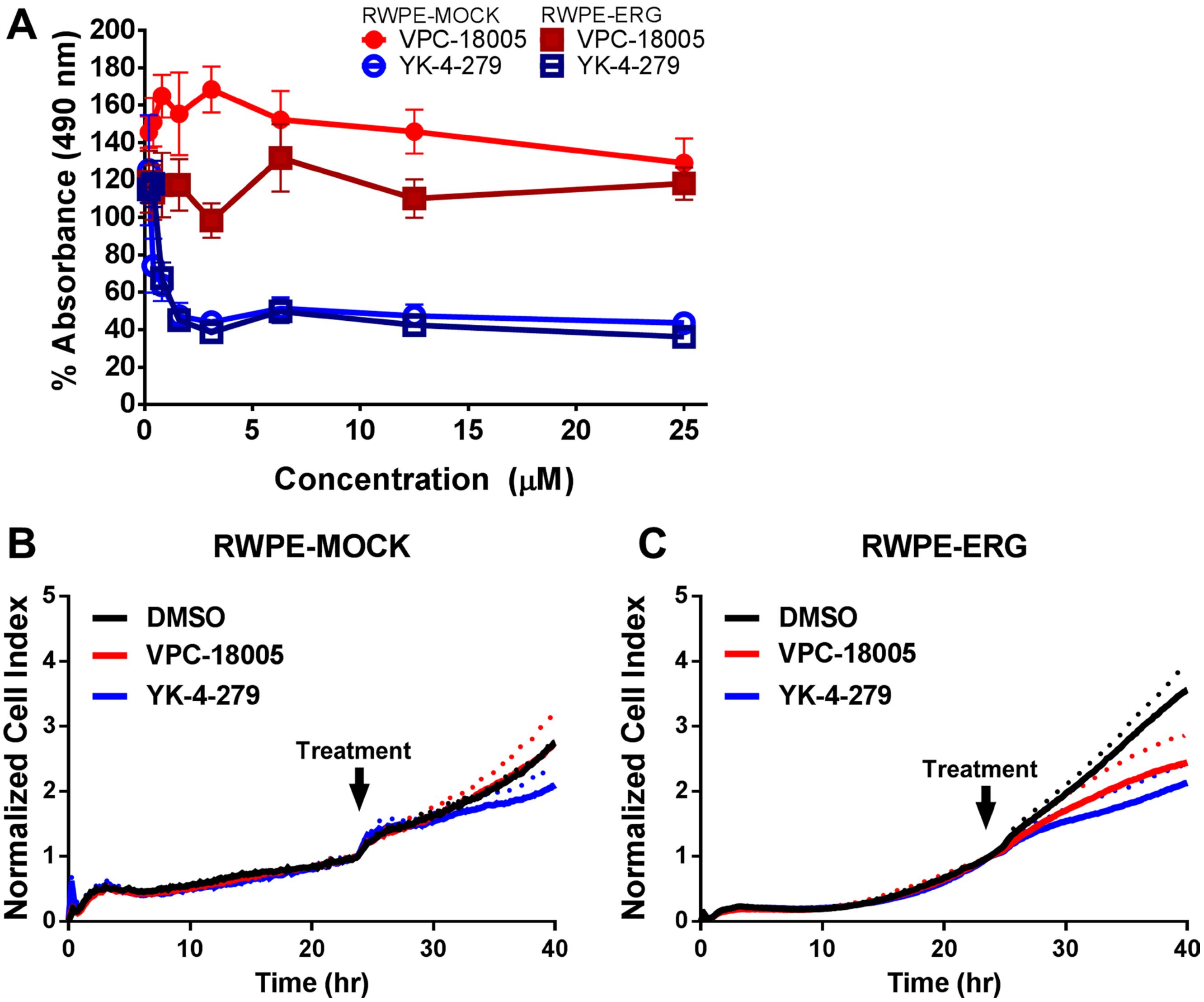

Supplementary Figure 4: VPC-18005 suppresses migration, but not growth of RWPE-1-ERG cells. (**A**) Cell viability (MTS) of ERG-expressing cells (RWPE-ERG) (closed circle) and non-ERG expressing cells (RWPE-MOCK (open square)) after treatment with 0.2 to 25 µM VPC-18005 (red) or published inhibitor YK-4-279 (blue) for 72 h. Impact on viability is presented as the mean ± SEM of 3 technical replicates and expressed as a percentage of absorbance at 490 nm relative to DMSO control. (**B**) RWPE-1-Mock or (**C**) RWPE-1-ERG cells were seeded in the upper chamber of a real-time cell analysis system (xCelligence) and treated with vehicle (0.01% DMSO, black), or 5 µM VPC-18005 (red) or YK-4-279 (blue) for 24 h . The normalized cell index is a measure of the migration of the cells through the pores of the upper chamber and is used as the migration index. Dotted lines represent 1 standard deviation (SD) of the mean migration rate.

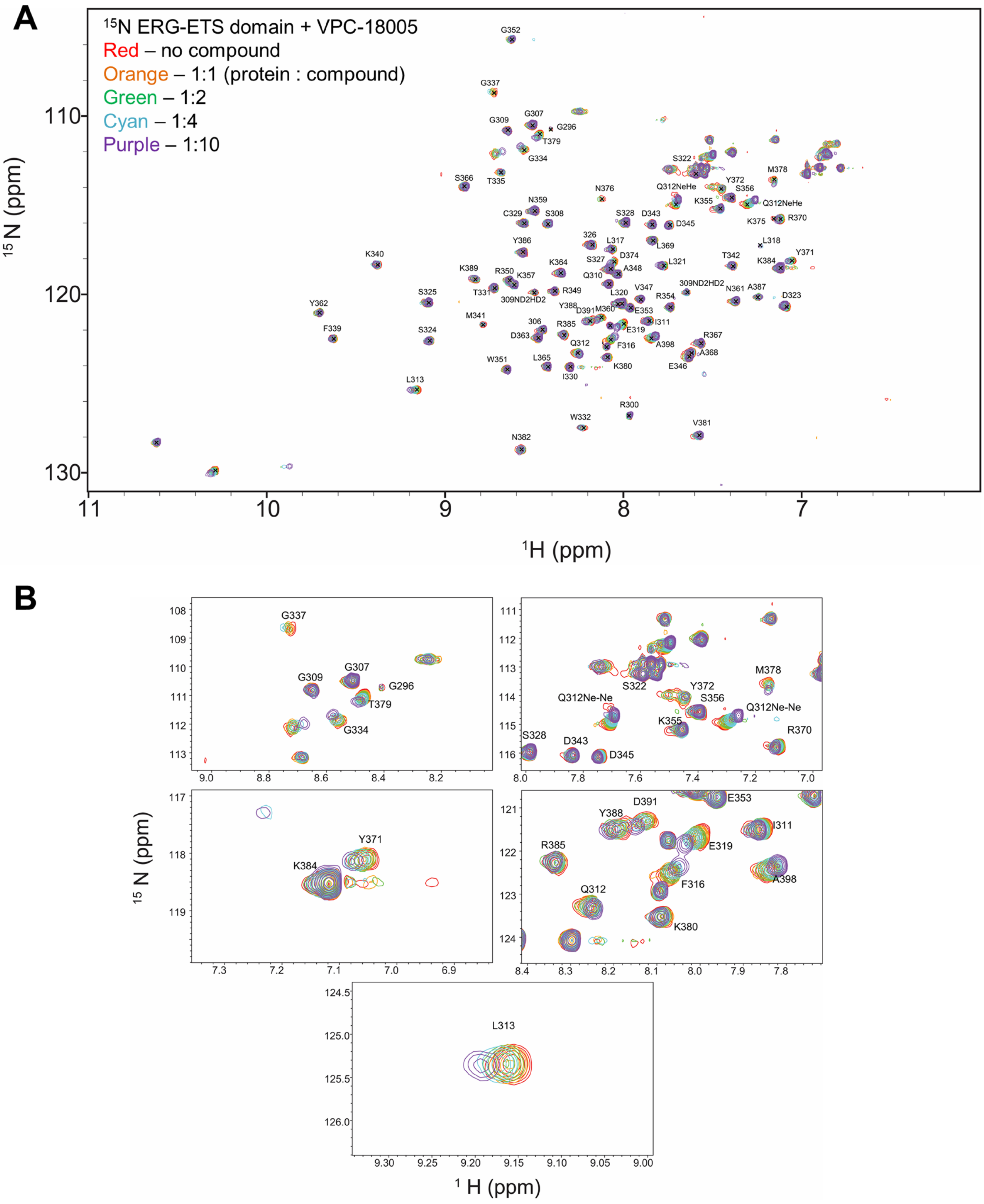
A
15N ERG-ETS domain + VPC-18005
Red – no compound
Orange – 1:1 (protein : compound)
Green – 1:2
Cyan – 1:4
Purple – 1:10
15 N (ppm)
1H (ppm)
B
15 N (ppm)
1 H (ppm)

C

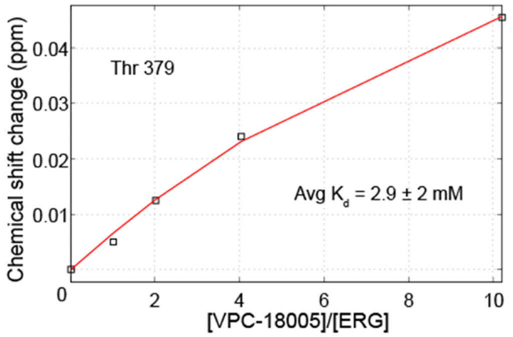

D

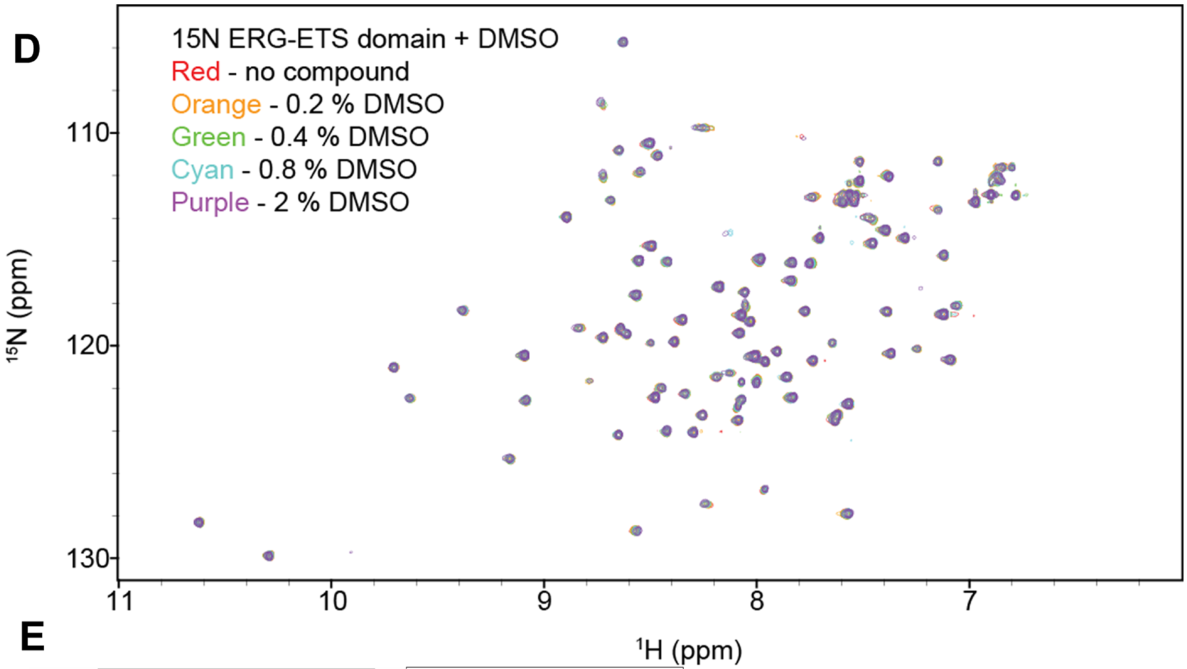

E

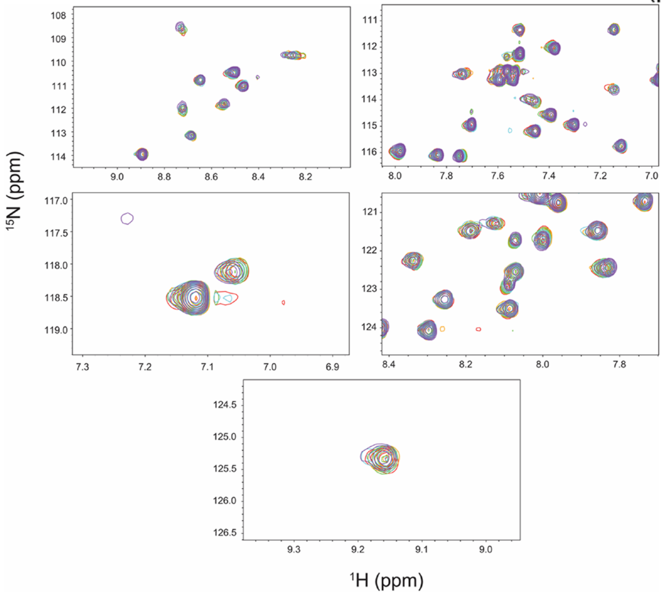

Supplementary Figure 5: VPC-18005 directly binds to the ERG-ETS domain. (**A**) Overlaid ^{15}N-HSQC spectra of ERG-ETS domain (100 μM) in the absence (red) and presence of increasing protein:compound molar ratios of DMSO-solubilized VPC-18005 (orange 1:1, green 1:2, cyan 1:4, and purple 1:10). (**B**) Expanded regions of the overlaid spectra. (**C**) Fitting of the VPC-18005-induced chemical shift perturbations of the amide $^{1}H^{N}$-^{15}N signals of residues 319, 323, 334, 371, and 379 (shown) to a simple 1:1 binding isotherm yielded an average K_D ~ 3 mM. (**D**) Overlaid ^{15}N-HSQC spectra of ERG-ETS domain (100 μM) in the absence (red) and presence of increasing concentration of DMSO (orange 0.2%, green 0.4%, cyan 0.8%, and purple 2%). (**E**) Expanded regions of the overlaid spectra, showing no perturbations due to the control titration with DMSO.

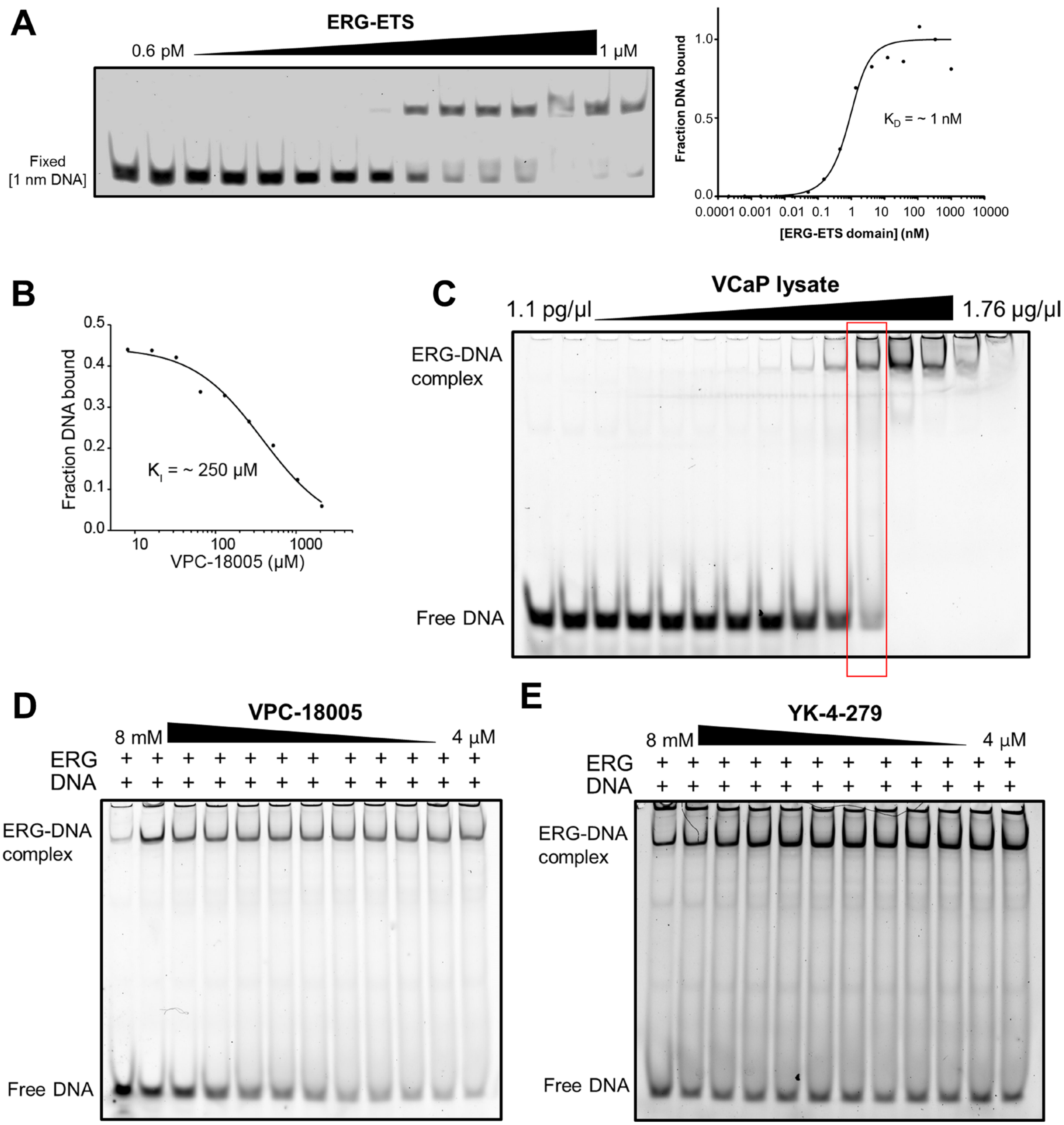

Supplementary Figure 6: VPC-18005 disrupts binding of the ERG-ETS domain to DNA. (**A**) EMSA analysis of the binding of from 0.6 pM to 1 mM of purified ERG-ETS domain to a fixed concentration of 1 nM fluorescently-labeled dsDNA was performed as detailed in manuscript Materials and Methods. Fitting of densitrometic analysis of free (lower band) and bound (upper band) DNA probe data to a 1:1 binding isotherm yielded a K_D value of ~ 1 nM. (**B**) Fitting of the data from Figure 3B (VPC-18005; middle panel) to simple competition isotherm yielded a K_I value of ~ 250 μM for the interaction of VPC-18005 with the ERG-ETS domain. (**C**) EMSA analysis of the binding of from 1.1 pg/μl to 1.76 μg/μl of VCaP nuclear lysate to a fixed concentration of 1 nM fluorescently-labeled dsDNA. EMSA analysis of the binding of 55 ng/μl ERG-ETS domain to 1 nM fluorescently-labeled dsDNA in the presence of 4 μM to 8 mM of (**D**) VPC-18005, and (**E**) YK-4-279.

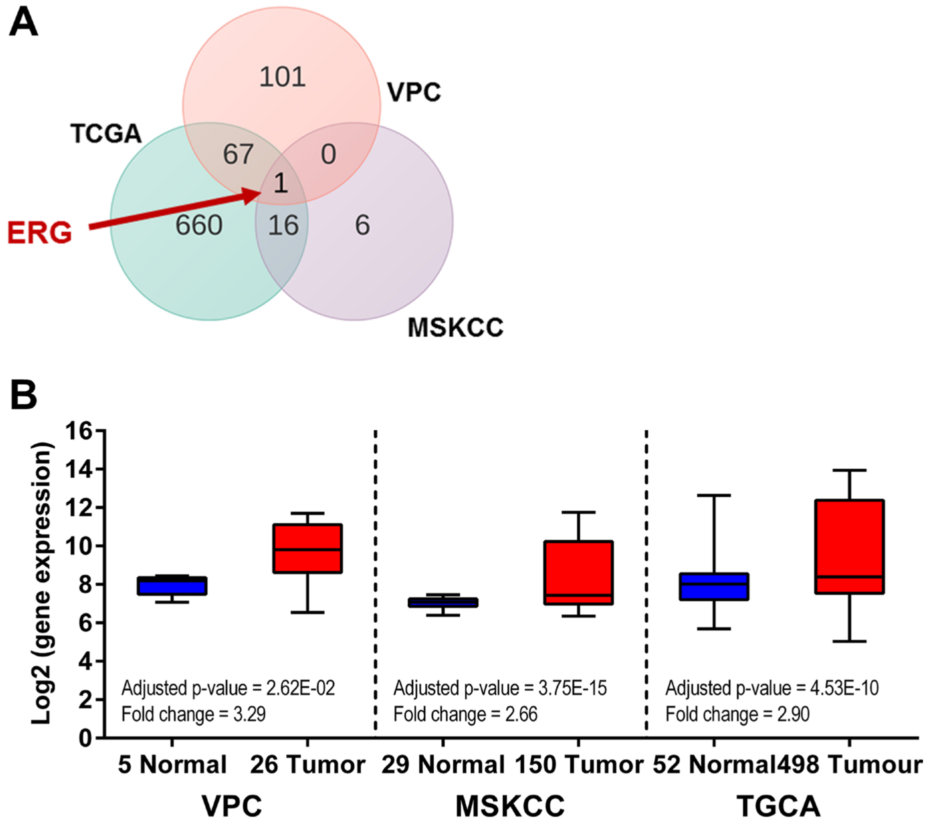

Supplementary Figure 7: ERG is overexpressed in prostate cancer. (**A**) A Venn diagram that shows the number of upregulated genes from each of the three gene expression datasets: Vancouver Prostate Centre (VPC), Memorial Sloan-Kettering Cancer Center (MSKCC), and The Cancer Genome Atlas (TCGA), based on a bioinformatic protocol (see Supplementary Materials and Methods). *ERG* is the only overexpressed gene common to the three datasets. (**B**) The fold changes of *ERG* gene expression in PCa tumour samples, compared to normal samples, range from 2.66 to 3.29.

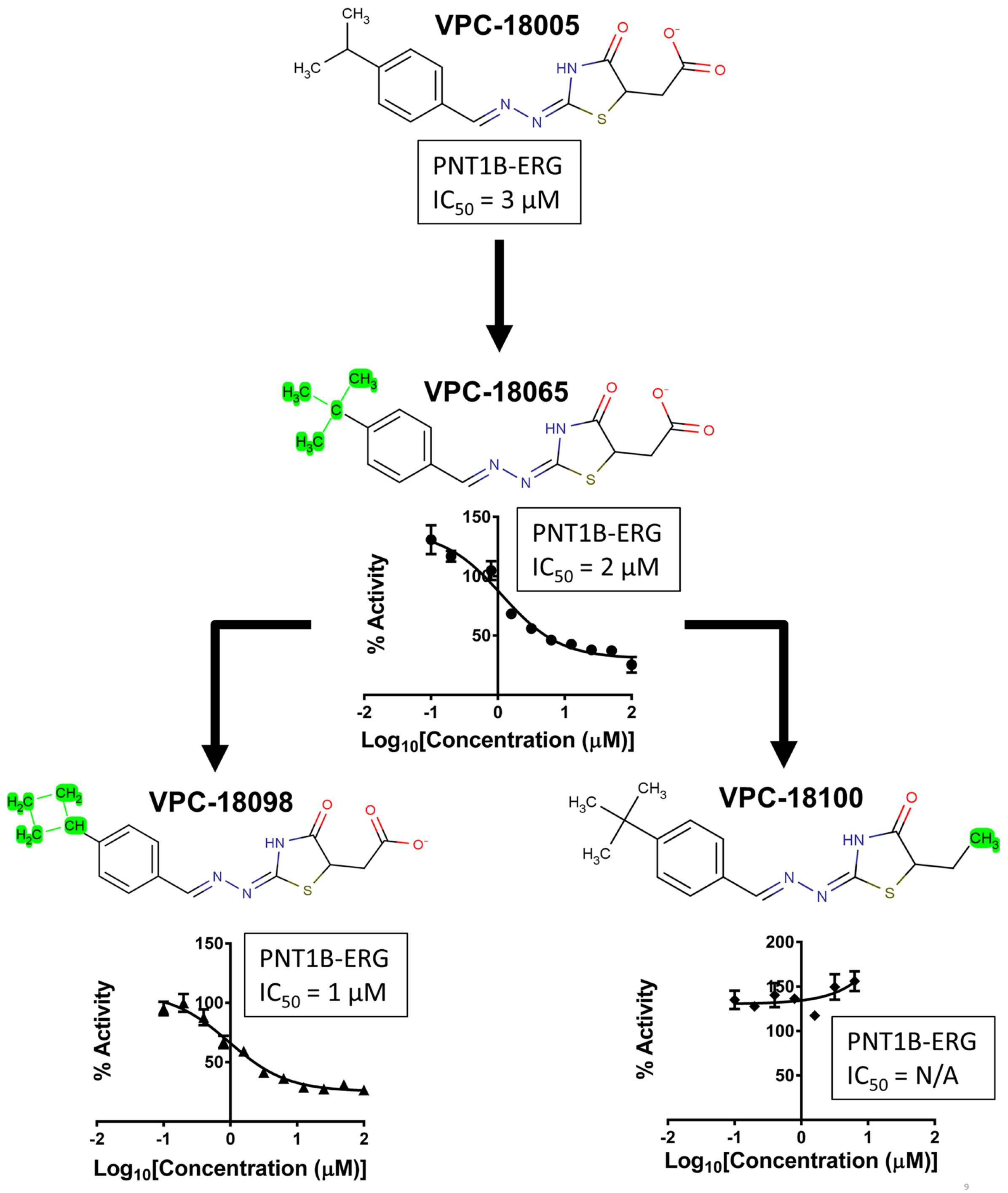

Supplementary Figure 8: Preliminary SAR studies using derivatives of VPC-18005. Modifications of the isopropyl moiety (VPC-18005) into tert-butyl (VPC-18065) and cyclobutyl (VPC-18098) improved the IC_{50} values in the luciferase reporter assays in PNT1B-ERG cells. Removal of the carboxyl moiety (VPC-18100) resulted in the loss of activity. Progressive differences between the derivatives are highlighted in green.

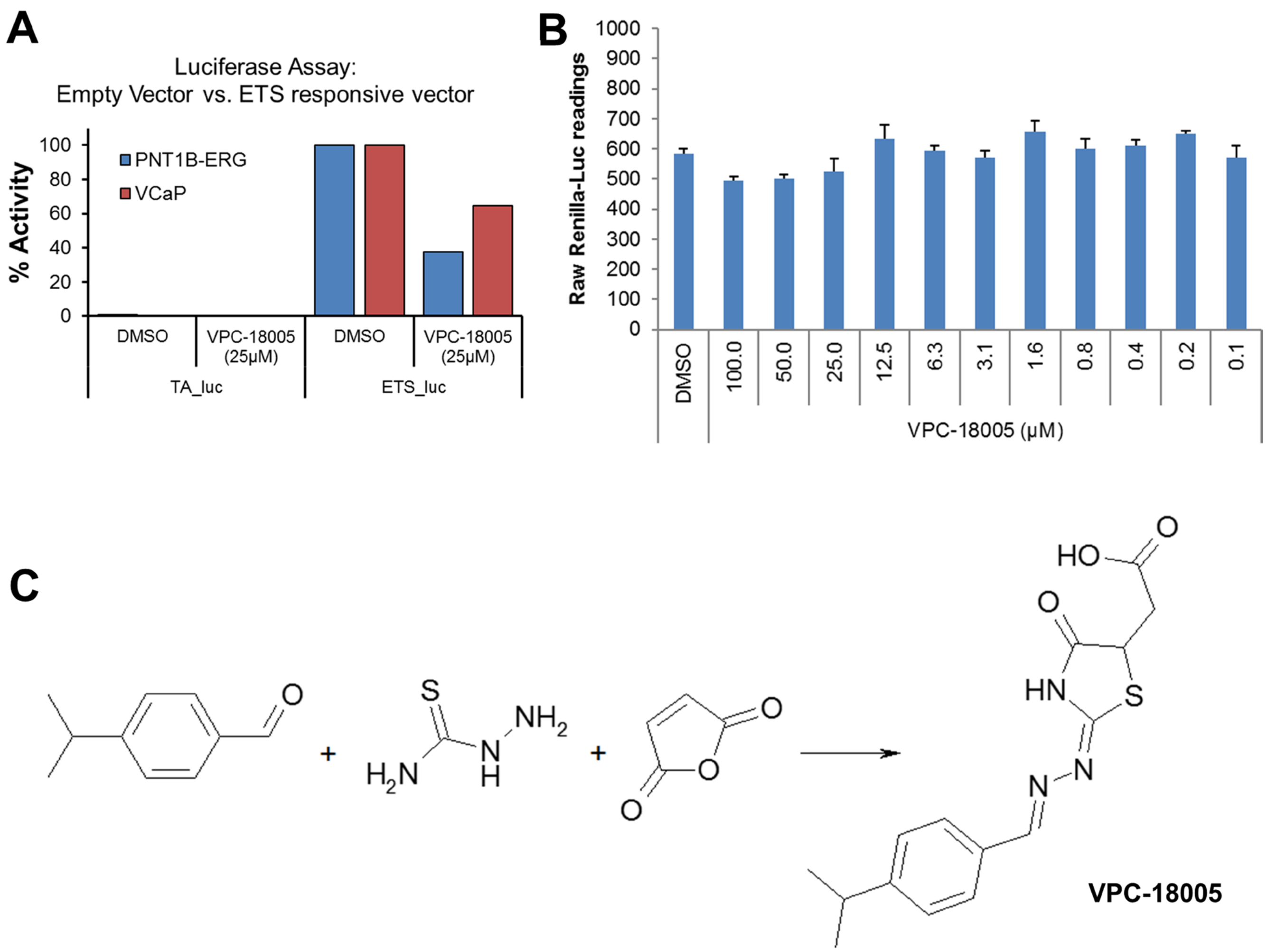

Supplementary Figure 9: (**A**) Comparison of empty vector vs. ETS responsive vector and the effect of DMSO control and VPC-18005 accordingly. Data points represent the mean of quadruplicate values. Error bars indicate standard error of mean for *n* = 4 values. (**B**) Raw renilla (pRL-tk) luciferase readings from a dose response experiment where PNT1B-ERG cells were treated with 0.1 – 100 µM of VPC-18005. (**C**) General scheme of chemical synthesis for VPC-18005.

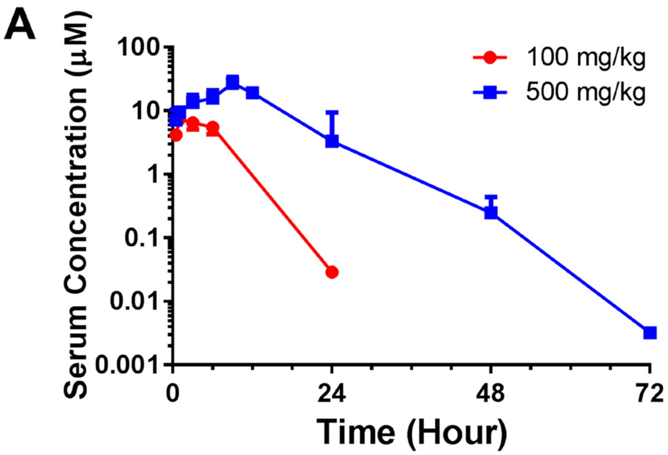

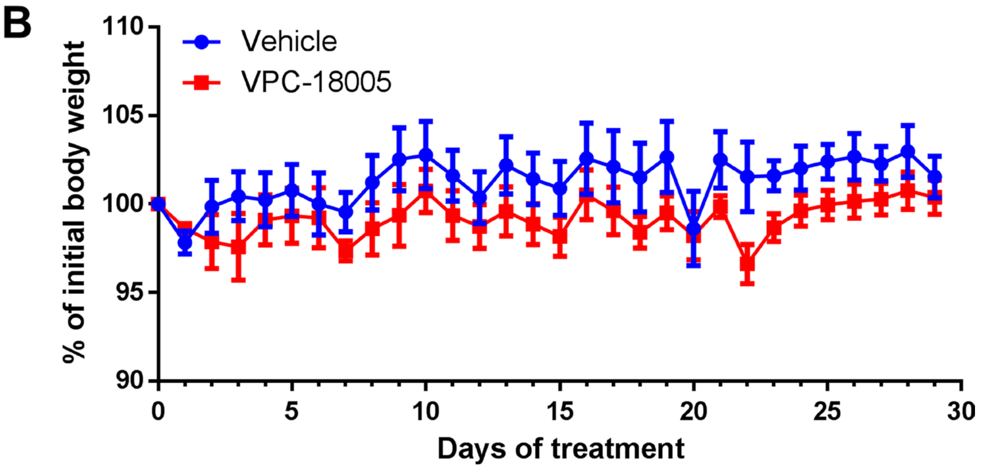

C

	Vehicle		VPC-18005		Paired t-test
Test	Avg Conc.	SD	Avg Conc.	SD	p-value
ALB (g/L)	40.75	1.48	40.00	2.45	0.6971
ALP (U/L)	31.50	8.29	38.00	6.52	0.2104
ALT (U/L)	49.33	2.05	36.25	2.77	0.2130
AMY (U/L)	1439.25	132.26	1357.00	143.62	0.1378
TBIL (umol/L)	4.25	0.43	4.25	0.43	1.0000
BUN (mmol/L)	7.88	0.62	6.75	1.21	0.3416

Supplementary Figure 10: (**A**) In a preliminary study to evaluate *in vivo* toxicity and establish working oral (PO) doses, Nu/nu mice (n = 4) received a single PO dose of VPC-18005 (100 mg/kg or 500 mg/kg) dissolved in cyclodextrin. Blood was collected directly after injection and at the times indicated. Serum concentration of VPC-18005 was determined for each time point. (**B**) In a 4 week "repeated dose" study, body weight was measured daily for mice (n = 4) that received a daily dose of VPC-18005 (150 mg/kg BID) dissolved 50% ethanol (PO) or 50% ethanol (Vehicle). Change in weight of each animal was normalized to starting pretreatment weight. (**C**) Cytotoxicity serum markers of vehicle and VPC-18005 treated animals described in B) were determined and presented as average (Avg. Conc.) and standard deviation (SD) with corresponding paired t-test p values. LC-MS analysis of serum indicators of major organ toxicity (albumin (ALB), alkaline phosphatase (ALP) and alanine aminotransferase (ALT) as indicators of liver dysfunction, amylase (AMY) to test for acute pancreatitis, total bilirubin (TBIL) to assess jaundice, and blood urea nitrogen (BUN) to assess kidney function from endpoint samples indicated no significant impact of VPC-18005 treatment on their levels.
