# Supplementary material for: Discovery and characterization of small molecules targeting the DNA-binding ETS domain of ERG in prostate cancer
Source: Oncotarget. 2017 Apr 15;8(26):42438–54. doi: 10.18632/oncotarget.17124 (PMC5522078; doi:10.18632/oncotarget.17124)
Supplement: Supplementary file 2 [file oncotarget-08-42438-s002.docx]

**Supplementary Table 1.** Overexpressed genes common in the VPC and TCGA gene expression sets.

| **Gene Symbol** | **NCBI Gene ID** | **P-value (VPC)** | **Adjusted P-value (VPC)** | **Fold change (VPC)** | **P-value (TCGA)** | **Adjusted P-value (TCGA)** | **Fold change (TCGA)** |
| --- | --- | --- | --- | --- | --- | --- | --- |
| ACRV1 | 56 | 2.52E-04 | 2.64E-02 | 3.46 | 3.94E-05 | 1.03E-04 | 2.49 |
| ANGPTL3 | 27329 | 6.15E-04 | 4.33E-02 | 3.55 | 2.40E-12 | 2.19E-11 | 3.61 |
| ASF1B | 55723 | 3.04E-05 | 8.37E-03 | 2.89 | 2.10E-10 | 1.32E-09 | 2.21 |
| B4GALNT4 | 338707 | 3.85E-07 | 8.52E-04 | 2.87 | 2.81E-21 | 2.32E-19 | 3.39 |
| BARX1 | 56033 | 2.42E-04 | 2.58E-02 | 6.22 | 3.80E-13 | 4.15E-12 | 2.68 |
| BUB1 | 699 | 1.89E-05 | 6.41E-03 | 2.91 | 9.15E-11 | 6.12E-10 | 2.74 |
| BUB1B | 701 | 3.31E-04 | 3.11E-02 | 3.32 | 7.82E-15 | 1.24E-13 | 3.54 |
| C17orf55 | 284185 | 7.88E-04 | 4.95E-02 | 2.80 | 3.65E-07 | 1.31E-06 | 2.16 |
| CCNA2 | 890 | 2.59E-06 | 2.88E-03 | 2.38 | 1.10E-13 | 1.34E-12 | 2.63 |
| CDC20 | 991 | 6.08E-05 | 1.29E-02 | 2.88 | 7.24E-15 | 1.16E-13 | 3.57 |
| CDC25C | 995 | 2.91E-06 | 3.05E-03 | 3.79 | 3.12E-15 | 5.49E-14 | 4.93 |
| CDC45 | 8318 | 7.08E-04 | 4.68E-02 | 2.65 | 1.66E-10 | 1.06E-09 | 3.66 |
| CDCA3 | 83461 | 1.98E-04 | 2.32E-02 | 3.37 | 2.13E-14 | 3.06E-13 | 2.34 |
| CDKN3 | 1033 | 2.19E-05 | 6.91E-03 | 2.64 | 2.26E-11 | 1.70E-10 | 3.39 |
| CELSR3 | 1951 | 5.26E-04 | 3.97E-02 | 2.67 | 2.46E-13 | 2.79E-12 | 2.74 |
| COMP | 1311 | 8.56E-05 | 1.41E-02 | 6.10 | 3.20E-11 | 2.34E-10 | 6.66 |
| CPLX1 | 10815 | 4.23E-04 | 3.44E-02 | 3.52 | 8.90E-22 | 8.23E-20 | 2.54 |
| CPNE7 | 27132 | 1.63E-05 | 5.80E-03 | 5.27 | 2.14E-09 | 1.11E-08 | 4.29 |
| DLGAP5 | 9787 | 7.49E-04 | 4.82E-02 | 6.64 | 1.19E-12 | 1.15E-11 | 5.15 |
| EPHA8 | 2046 | 1.48E-05 | 5.67E-03 | 4.28 | 4.29E-16 | 9.32E-15 | 4.48 |
| ERG | 2078 | 2.47E-04 | 2.62E-02 | 3.29 | 6.60E-11 | 4.53E-10 | 2.90 |
| ESPL1 | 9700 | 2.20E-06 | 2.88E-03 | 3.47 | 3.99E-11 | 2.87E-10 | 2.43 |
| FAM57B | 83723 | 3.84E-05 | 9.36E-03 | 7.06 | 5.44E-10 | 3.17E-09 | 2.41 |
| FAM64A | 54478 | 6.11E-04 | 4.33E-02 | 3.65 | 6.45E-13 | 6.65E-12 | 3.99 |
| GTSE1 | 51512 | 5.97E-04 | 4.25E-02 | 3.64 | 5.34E-14 | 7.01E-13 | 4.28 |
| HJURP | 55355 | 2.53E-06 | 2.88E-03 | 4.09 | 8.10E-16 | 1.65E-14 | 6.22 |
| IQGAP3 | 128239 | 7.06E-04 | 4.68E-02 | 3.48 | 3.54E-15 | 6.15E-14 | 4.20 |
| KIF4A | 24137 | 9.83E-06 | 5.15E-03 | 4.86 | 2.23E-14 | 3.20E-13 | 5.69 |
| KIFC1 | 3833 | 1.59E-04 | 2.02E-02 | 2.80 | 9.58E-13 | 9.50E-12 | 2.77 |
| KLHL35 | 283212 | 1.57E-04 | 2.01E-02 | 3.84 | 2.41E-19 | 1.20E-17 | 2.92 |
| LRRC36 | 55282 | 9.70E-06 | 5.15E-03 | 2.25 | 6.28E-09 | 3.01E-08 | 2.40 |
| MGAT5B | 146664 | 2.48E-05 | 7.41E-03 | 3.39 | 2.61E-12 | 2.36E-11 | 3.18 |
| MLF1IP | 79682 | 2.71E-05 | 7.68E-03 | 2.41 | 4.26E-10 | 2.53E-09 | 2.19 |
| MYBL2 | 4605 | 5.98E-06 | 3.95E-03 | 4.50 | 2.08E-13 | 2.40E-12 | 6.21 |
| NCAPG | 64151 | 1.32E-05 | 5.36E-03 | 3.39 | 2.09E-12 | 1.93E-11 | 3.87 |
| NEIL3 | 55247 | 1.95E-05 | 6.44E-03 | 4.77 | 2.59E-16 | 5.87E-15 | 4.01 |
| NEK2 | 4751 | 6.73E-06 | 4.35E-03 | 5.11 | 2.77E-12 | 2.49E-11 | 3.95 |
| NETO2 | 81831 | 1.17E-05 | 5.36E-03 | 6.52 | 1.21E-23 | 1.69E-21 | 4.04 |
| NKX2-2 | 4821 | 8.49E-05 | 1.41E-02 | 12.60 | 7.84E-09 | 3.71E-08 | 3.26 |
| NKX6-1 | 4825 | 2.59E-06 | 2.88E-03 | 18.08 | 8.57E-20 | 4.78E-18 | 2.84 |
| NR2E1 | 7101 | 4.83E-05 | 1.10E-02 | 2.97 | 7.77E-14 | 9.88E-13 | 2.08 |
| OIP5 | 11339 | 6.78E-04 | 4.55E-02 | 2.22 | 1.84E-08 | 8.12E-08 | 2.01 |
| ONECUT2 | 9480 | 5.46E-04 | 4.06E-02 | 5.99 | 1.57E-16 | 3.77E-15 | 8.80 |
| PBK | 55872 | 6.36E-04 | 4.42E-02 | 2.78 | 2.54E-11 | 1.89E-10 | 3.57 |
| PLK1 | 5347 | 2.52E-04 | 2.64E-02 | 2.07 | 3.03E-16 | 6.78E-15 | 3.39 |
| PPP1R14B | 26472 | 1.59E-05 | 5.74E-03 | 4.36 | 4.07E-17 | 1.16E-15 | 2.43 |
| PRC1 | 9055 | 3.02E-06 | 3.05E-03 | 2.51 | 6.91E-14 | 8.86E-13 | 2.01 |
| PTPRT | 11122 | 3.75E-04 | 3.26E-02 | 6.51 | 2.43E-06 | 7.67E-06 | 3.14 |
| RAB19 | 401409 | 4.54E-04 | 3.61E-02 | 4.02 | 9.23E-09 | 4.29E-08 | 2.12 |
| RDM1 | 201299 | 2.62E-05 | 7.58E-03 | 6.57 | 4.14E-10 | 2.47E-09 | 2.35 |
| RNFT2 | 84900 | 3.55E-05 | 8.97E-03 | 2.16 | 2.74E-10 | 1.69E-09 | 2.32 |
| RRM2 | 6241 | 3.38E-04 | 3.14E-02 | 4.06 | 1.56E-12 | 1.48E-11 | 4.42 |
| SHCBP1 | 79801 | 2.19E-04 | 2.48E-02 | 3.38 | 2.30E-10 | 1.43E-09 | 2.81 |
| SPAG5 | 10615 | 2.59E-04 | 2.66E-02 | 2.02 | 1.96E-14 | 2.85E-13 | 2.50 |
| SPC24 | 147841 | 3.43E-04 | 3.14E-02 | 2.31 | 2.42E-13 | 2.75E-12 | 3.05 |
| SRCIN1 | 80725 | 3.88E-04 | 3.32E-02 | 2.26 | 1.47E-08 | 6.62E-08 | 2.06 |
| TK1 | 7083 | 2.28E-06 | 2.88E-03 | 2.45 | 1.76E-12 | 1.65E-11 | 2.24 |
| TOP2A | 7153 | 5.07E-05 | 1.13E-02 | 2.78 | 3.34E-10 | 2.03E-09 | 3.05 |
| TPX2 | 22974 | 6.81E-05 | 1.33E-02 | 3.27 | 1.79E-13 | 2.09E-12 | 3.49 |
| TRIP13 | 9319 | 1.45E-04 | 1.93E-02 | 2.84 | 3.81E-12 | 3.33E-11 | 2.65 |
| TROAP | 10024 | 7.58E-05 | 1.36E-02 | 4.77 | 5.09E-16 | 1.08E-14 | 4.45 |
| TTLL6 | 284076 | 1.08E-04 | 1.61E-02 | 2.58 | 4.24E-13 | 4.59E-12 | 2.23 |
| UBE2C | 11065 | 6.92E-04 | 4.60E-02 | 5.45 | 4.86E-13 | 5.16E-12 | 4.86 |
| UGT2B4 | 7363 | 4.24E-04 | 3.44E-02 | 7.18 | 2.32E-14 | 3.30E-13 | 8.55 |
| WDR62 | 284403 | 1.52E-04 | 1.98E-02 | 2.71 | 6.52E-09 | 3.13E-08 | 2.17 |
| ZIC2 | 7546 | 6.39E-07 | 1.11E-03 | 25.22 | 1.21E-22 | 1.32E-20 | 24.17 |
| ZIC5 | 85416 | 2.19E-04 | 2.48E-02 | 7.87 | 8.79E-26 | 2.26E-23 | 10.42 |
| ZP3 | 7784 | 1.42E-04 | 1.92E-02 | 2.18 | 2.74E-12 | 2.47E-11 | 2.33 |
